# Extracellular Vesicle Proteome Analysis Improves Diagnosis of Recurrence in Triple‐Negative Breast Cancer

**DOI:** 10.1002/jev2.70089

**Published:** 2025-06-23

**Authors:** Ju‐Yong Hyon, Min Woo Kim, Kyung‐A Hyun, Yeji Yang, Seongmin Ha, Jee Ye Kim, Young Kim, Sunyoung Park, Hogyeong Gawk, Heaji Lee, Suji Lee, Sol Moon, Eun Hee Han, Jin Young Kim, Ji Yeong Yang, Hyo‐Il Jung, Seung Il Kim, Young‐Ho Chung

**Affiliations:** ^1^ Research Center for Digital Omics Korea Basic Science Institute Cheongju Republic of Korea; ^2^ Department of Surgery Yonsei University College of Medicine Seoul Republic of Korea; ^3^ School of Biopharmaceutical and Medical Sciences Sungshin Women's University Seoul Republic of Korea; ^4^ Critical Diseases Diagnostics Convergence Research Center Korea Research Institute of Bioscience and Biotechnology Daejeon Republic of Korea; ^5^ Department of Biological Sciences Korea Advanced Institute for Science and Technology (KAIST) Daejeon Republic of Korea; ^6^ School of Mechanical Engineering Yonsei University Seoul Republic of Korea; ^7^ The DABOM Inc. Seoul Republic of Korea; ^8^ Biopharmaceutical Research Center Korea Basic Science Institute Cheongju Republic of Korea; ^9^ Department of Bio‐Analytical Science University of Science and Technology Daejeon Republic of Korea; ^10^ Department of Analytical Science and Technology, Graduate School of Analytical Science and Technology (GRAST) Chungnam National University Daejeon Republic of Korea

**Keywords:** diagnosis, machine learning, microfluidics, proteomic analysis, triple‐negative breast cancer, tumour derived extracellular vesicles

## Abstract

We explored the diagnostic utility of tumor‐derived extracellular vesicles (tdEVs) in breast cancer (BC) by performing comprehensive proteomic profiling on plasma samples from 130 BC patients and 40 healthy controls (HC). Leveraging a microfluidic chip‐based isolation technique optimized for low plasma volume and effective contaminant depletion, we achieved efficient enrichment of tdEVs. Proteomic analysis identified 26 candidate biomarkers differentially expressed between BC patients and HCs. To enhance biomarker selection robustness, we implemented a hybrid machine learning framework integrating LsBoost, convolutional neural networks, and support vector machines. Among the identified candidates, four EV proteins. ECM1, MBL2, BTD, and RAB5C. not only exhibited strong discriminatory performance, particularly for triple‐negative breast cancer (TNBC), but also demonstrated potential relevance to disease recurrence, providing prognostic insights beyond initial diagnosis. Receiver operating characteristic (ROC) curve analysis demonstrated high diagnostic accuracy with an area under the curve (AUC) of 0.924 for BC and 0.973 for TNBC, as determined by mass spectrometry. These findings were further substantiated by immuno assay validation, which yielded an AUC of 0.986 for TNBC. Collectively, our results highlight the potential of EV proteomics as a minimally invasive, blood‐based platform for both accurate detection and recurrence risk stratification in breast cancer and its aggressive subtypes, offering promising implications for future clinical applications.

## 1 Background

Cancer is a complex mosaic of diverse cells of varying sizes, distinct genetic profiles and unique molecular characteristics within the tumour environment (Fisher et al. 2013). This concept, known as tumour heterogeneity, not only adds layers of complexity to cancer biology, but also influences the critical processes of carcinogenesis, metastasis, acquired resistance, pre‐metastatic niche formation and angiogenesis, thereby impacting patient prognosis (Dagogo‐Jack and Shaw 2018; Bedard et al. 2013; Lawson et al. 2018). Recently, advanced sequencing techniques and other ‘omics’ studies have revealed that different levels of heterogeneity exist across cancer types (Bianchini et al. 2016). For instance, breast cancer (BC) has been commonly categorized into four main subtypes, including luminal A, luminal B, HER2‐enriched and triple‐negative breast cancer (TNBC), based on genetic and molecular characteristics. However, in comprehensive genetic studies, Lehmann et al. classified TNBC into six distinct subtypes, adding complexity to its diagnosis and prognosis (Lehmann et al. 2011, 2016). They revealed a notable level of heterogeneity in TNBC compared with other subtypes.

Tumour heterogeneity in TNBC is associated with rapid tumour growth, high metastatic potential and poor clinical outcomes (Zagami and Carey 2022). Clinically, patients with TNBC often have increased recurrence and metastasis rates, leading to shorter 5‐year survival rates and shorter overall lifespans (Baranova et al. 2022). Despite a relatively low incidence of 10%–15% among BC subtypes, the aggressive nature of TNBCs emphasizes the need to find reliable diagnostic methods with strong diagnostic power for patients with TNBC and those with recurrent TNBC. In this study, we aimed to improve TNBC diagnostic power using accurate protein signatures of tumour‐derived EV (tdEV), beyond simply detecting BC regardless of subtype. During this process, the quality and accuracy of sample collection are crucial for the integrity of omics‐based biomarker discovery and diagnosis. Typically, pathologists rely on light microscopy to observe tumour cross sections immuno‐labelled with specific antibodies. However, this piecemeal approach may fail to reflect the three‐dimensional structure of the tumour and create a discrepancy between the derived data and the original tumour state (Tanaka et al. 2017). It is essential to understand that the procedure for obtaining tumour samples through needle aspiration or surgery for in‐depth analysis can inevitably compromise cellular spatial localization, thus potentially missing the overall genetic and proteomic landscape of the tumour. Hence, liquid biopsies are promising alternatives in the recent era of precision medicine because of their noninvasive and repeatable nature (Ignatiadis et al. 2021; Heitzer et al. 2019).

Biomarker discovery in liquid biopsies is extensive, with molecules such as circulating tumour DNA (ctDNA), circulating tumour cells (CTC), microRNAs (miRNAs) and extracellular vesicles (EVs). Recently, EVs have gained notable attention, particularly for understanding cancer growth, drug resistance and metastatic behaviour (Kalluri 2016). Notably, several in‐depth studies on EV proteome profiling have found that key oncogenic proteins are abundant in EVs isolated from the peripheral blood of patients with BC (Chen et al. 2017; Moon et al. 2016; Lee et al. 2016; Tutanov et al. 2020; Li et al. 2021). EVs contain a diverse range of proteins, reflect cellular dynamics and surpass secreted proteins in terms of quantity and stability (Li et al. 2015). However, mass spectrometry for proteomic analysis provides an unbiased screen for numerous proteins present in heterogeneous populations of EVs, possibly missing low‐abundance proteins present in tumour‐derived EV (tdEV) (Kowal et al. 2016; Rontogianni et al. 2019). To address this shortcoming, we employed a microfluidic chip, a sensitive and cost‐effective tool, to determine the protein profiles of tdEVs from patients with TNBC by targeting and concentrating tdEVs with BC‐derived proteins expressed on EVs, such as epithelial cell adhesion molecule (EpCAM) and CD49f (Gwak et al. 2021, 2022). In our previous study, CD49f and EpCAM biomarkers were found to be highly expressed in EVs derived from four cell lines (Luminal A for MCF‐7, Luminal B for BT‐474, HER‐2 for SK‐BR‐3 and TNBC for MDA‐MB‐231), reflecting subtypes of breast cancer. This microfluidic approach offers several advantages. First, it reduces interference from abundant proteins such as albumin and immunoglobulin and ensures enhanced sample purity, even in limited quantities (Contreras‐Naranjo et al. 2017, 2018). Additionally, the microfluidic chip was designed to isolate tdEVs, which constitute less than 1% of the total EVs (D'Souza‐Schorey and Clancy 2012; Whiteside 2016). Furthermore, the lipid bilayer of EVs protects proteins from degradation, offering a stability over other blood proteins.

Interpreting liquid biopsy data using traditional analytical methods can be challenging because of the noise, complexity, and biological sample variability. However, since the beginning of the 21st century, advances in machine learning (ML) have offered the potential to derive clarity (Richens et al. 2020; Kononenko 2001; Kleppe et al. 2021). By navigating numerous datasets with complex characteristics, optimal ML algorithms can enhance diagnostic precision and pinpoint disease‐associated patterns.

**FIGURE 1:**
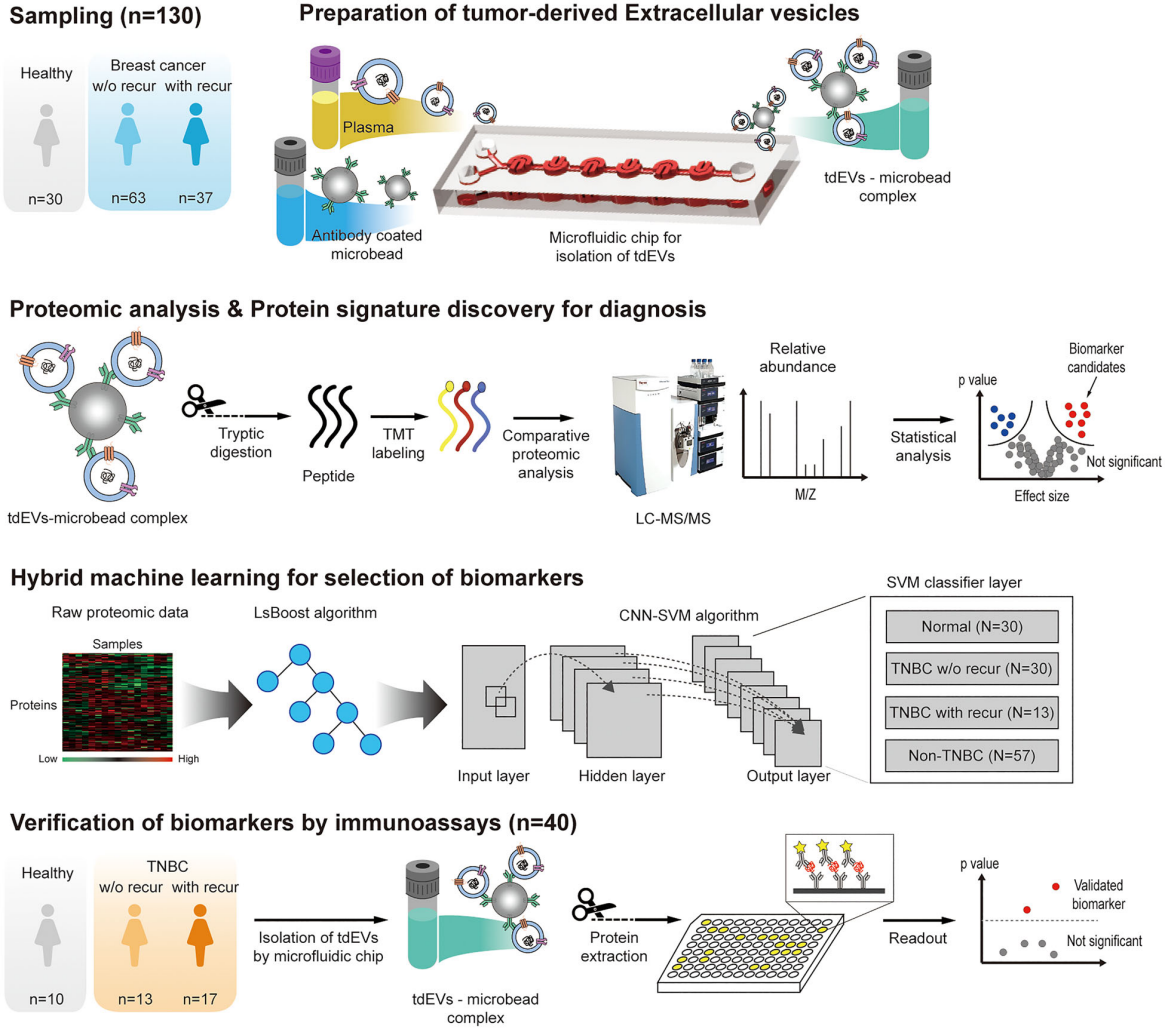
Schematic overview of the workflow for biomarker discovery by using proteomics with tdEVs to predict subtypes and the probability of BC recurrence. Three groups of patients, including normal, BC w/o recur and BC with recur, were recruited and asked to provide plasma samples. The samples were injected into a microfluidic chip with a BC‐related antibody (EpCAM and CD49f) coated microbead solution. The microfluidic chip produced tdEVs‐microbead complexes within 1 min based on immunoaffinity. Proteomic analysis by LC‐MS/MS using a tandem mass tag system identified signature proteins of tdEV that could be used to predict BC recurrence. A hybrid ML algorithm (LsBoost‐CNN‐SVM) was introduced to select biomarkers. The intensities of each protein were regressed by the LsBoost algorithm, and the data from LsBoost were classified using the CNN‐SVM hybrid algorithm. The selected four tdEV biomarkers were validated with plasma samples from 10 normal volunteers and 30 TNBC patients. The tdEVs prepared by the microfluidic chip were analysed using a conventional ELISA. BC, breast cancer; CNN, convolutional neural network; ELISA, enzyme‐linked immunosorbent assay; SVM, support vector machine; tdEVs, tumour‐derived EVs; with recur, with recurrence; w/o recur, without recurrence.

In this study, we combined the precision of microfluidics‐based isolation with the analytical power of ML to develop a hybrid algorithm for identifying an enhanced EV protein signature in BC (Figure 1). Considering these advantages, this synergistic approach facilitates a faster, more accurate, and minimally invasive cancer diagnosis. The identified EV protein signature could effectively distinguish patients with BC from healthy controls (HC). In particular, the discrimination ability using this signature could be more strongly applied not only to patients with TNBC but also to those at risk of recurrence. Furthermore, the relevance of TNBC patients’ overall and progression‐free survival rates could be assessed. In summary, our study sought to evaluate and emphasize the exceptional diagnostic accuracy of the EV protein signature when identifying BC, including the aggressive TNBC.

## 2 Materials and Methods

### 2.1 Study Design

We obtained written informed consent for the use of plasma samples for research purposes from all participants. We obtained clinical samples from subjects who visited Severance Hospital in South Korea, according to the guidelines of the independent Ethics Committee at the College of Medicine, Yonsei University (IRB No. 4‐2020‐0350, approved on 4 January 2021). This study was performed in accordance with the principles of the Declaration of Helsinki. The proteomic analysis involved 100 BC patients and 30 HC, with a similar average age, while the enzyme‐linked immunosorbent assay (ELISA) analysis included 30 TNBC patients and 10 HC. We collected preoperative plasma samples from the participants before anaesthesia. The inclusion criterion for healthy female control donors was a negative medical history of malignant disease. The criteria for patients with BC were as follows: (1) all blood samples were collected before any treatments, (2) the pathological diagnosis of BC was confirmed, and (3) haemolysis was assessed before the isolation of EVs to evaluate the quality of the plasma sample. To detect any recurrence signs, BC patients underwent follow‐up for a median period of 5 years post‐surgery, typically revisiting every 6 months for treatment efficacy evaluation and early recurrence (within 2 years) detection. Our criteria for recurrence were based on clinical examinations and mammograms to monitor for any signs of cancer.

### 2.2 Plasma Sample Collection

We collected blood samples in EDTA tubes and centrifuged them at 1500 × *g* for 15 min. We then collected the supernatant (plasma), which was stored at −80°C as a source for EV. Comprehensive clinical data for each participant were retrospectively collected, encompassing key characteristics such as sex, age, Ki‐67 status, conventional tumour marker status, subtypes, presence of metastasis, occurrence of recurrence, mortality, and staging according to the American Joint Committee on Cancer and TNM staging systems. The participants in this study are summarized in Table S1.

### 2.3 Cell Lines and Cell Cultures

We purchased BC cell lines MCF7, BT‐474, SK‐BR‐3, MDA‐MB‐231, and Hs578T from the American Type Culture Collection (ATCC; Manassas, VA, USA), and non‐tumorigenic epithelial cell lines, including human mammary epithelial cells (HMEC) and MCF10a cells, from the ATCC. We cultured all cancer cell lines in Roswell Park Memorial Institute‐1640 medium (RPMI‐1640; 22400‐089, Gibco, Carlsbad, CA, USA) supplemented with 10% foetal bovine serum (FBS; 12483‐020, Gibco) and 1% penicillin‐streptomycin (15140‐122, Gibco) and maintained them in a humidified incubator at 37°C with 5% CO_2_. HMECs were grown in mammary epithelial cell complete medium (PCS‐600‐030, ATCC) supplemented with a mammary epithelial cell growth kit (PCS‐600‐040, ATCC), following ATCC‐recommended protocols. MCF10A cells were grown in MEBM Basal Medium (CC‐3151, Lonza (Basel, Switzerland) supplemented with a MEGM SingleQuots (CC‐4136, Lonza). We tested all the cell lines for *Mycoplasma* contamination.

### 2.4 Fabrication of the Microfluidic Chip

Conventional soft‐lithography techniques have been used to fabricate microfluidic chips. The microchannel‐patterned mould was produced by placing a mask with a transparent channel structure on a silicon wafer coated with a negative photoresist (SU‐8 3035, MicroChem Corp., USA), irradiating it with UV light, and removing the uncured photoresist. Subsequently, polydimethylsiloxane (PDMS) mixed with a curing agent was poured into the mould to construct a microfluidic channel. The PDMS replica from the mould was punched to create inlet and outlet holes and then combined with a glass slide through an oxygen plasma treatment. Detailed information regarding the entire procedure can be found in our previous reports (Gwak et al. 2021, 2022; Yu et al. 2023).

### 2.5 Preparation of Antibody‐Coated Microbeads

To immobilize antibodies on the microbead surfaces, we mixed 5 µg of biotinylated antibodies (ab79079; Abcam, Cambridge, UK, and ST60037BT; Stem Cell Technologies, Canada) with 1 mg of streptavidin‐coated polystyrene microbeads (SVP‐60‐5; Spherotech, USA). The mixture was incubated for 30 min at room temperature (23°C) using a rotator.

### 2.6 Microfluidic Chip for Isolation of EVs

We isolated tumour‐derived EVs (tdEVs) using a previously reported microfluidic chip (Kim et al. 2021). The 300 µL of plasma sample and 300 µL of a mixture of each anti‐EpCAM antibody and anti‐CD49f antibody conjugated to 7 µm microbeads (TheDABOM, Seoul, Republic of Korea) were injected into the chip. The microfluidic chip consisted of 150 cycles of a horseshoe‐shaped micromixer (HOMM) structure that generated fluidic folding and changes in the direction of the fluidic flow, enhancing collisions between tdEVs in the plasma and antibody‐coated microbeads. The tdEVs in 300 µL of plasma sample were captured onto the microbeads within 1 min and then harvested through the outlet of the chip.

### 2.7 Qualitative and Quantitative Characterization of EVs

EV characterization was performed using plasma samples from one representative individual per group: healthy control, non‐TNBC, TNBC patients without recurrence (TNBC w/o recur), and TNBC patients with recurrence (TNBC with recur). Nano tracking analysis (NTA; NanoSight NS300, Malvern Panalytical, UK) was used to determine size distribution and concentration, with triplicate measurements analysed using NTA software (v3.2). Scanning electron microscopy (SEM) confirmed EV morphology. tdEV‐attached microbeads were fixed with Karnovsky's fixative (2% glutaraldehyde and 2% paraformaldehyde in 0.1 M phosphate buffer, pH 7.4), washed with 0.1 M phosphate buffer, post‐fixed with 1% OsO₄, dehydrated with ethanol, platinum‐coated (Leica Microsystems ACE600, Germany), and imaged (MERLIN, Carl Zeiss AG, Germany) at ×20,000 magnification. Flow cytometry was used to validate the expression of tetraspanin markers (CD9, CD63, CD81) on tdEVs. Samples were stained with fluorescently conjugated antibodies (CD9‐PerCP, ab275669, Abcam, UK; CD81‐FITC, A15753, Invitrogen, Carlsbad, USA; CD63‐PE‐Cy7, 561982, BD Biosciences, San Jose, USA) for 1 h at 4°C, analysed using a BD FACS Fortessa (BD Biosciences, USA), and processed with FCS Express software (De Novo Software, Pasadena, USA).

### 2.8 PEG Precipitation Method for Isolating Extracellular Vesicles

We isolated total EVs using a total exosome isolation reagent (44844502; Thermo Fisher Scientific, Rockford, IL, USA). Plasma was centrifuged at 2000 × *g* for 10 min, and the supernatant was used. According to the manufacturer's protocol, the supernatants were mixed with a reagent equal to 1/2 the total volume of the supernatants and incubated at 4°C overnight. After centrifugation at 10,000 ×*g* for 1 h at 4°C, the supernatant was removed, and the pellet was re‐suspended in PBS and used for further analyses.

### 2.9 Sample Preparation for Proteomic Analysis

EVs isolated using the microfluidic chip were lysed with 1X sodium dodecyl sulphate (SDS) buffer (5% SDS, 50 mM triethylammonium bicarbonate (TEAB), pH 7.55). The protein extracts were reduced and alkylated with 20 mM dithiothreitol (DTT) and 40 mM indole‐3‐acetic acid (IAA). We performed tryptic digestion using an S‐Trap mini digestion kit (ProtiFi, Huntington, NY) following the manufacturer's protocol, and used mass‐spectrometry‐grade Trypsin Gold (Promega, Madison, WI, USA) for digestion at a protein to enzyme ratio of 10:1. The eluted samples were dried in speed vacuum and quantified using a Pierce Quantitative Colorimetric Peptide Assay Kit (Thermo Fisher Scientific). Next, we labelled 10 µg of trypsin‐digested peptides from each sample using 80 µg of 11‐plex TMT reagent (Thermo Fisher Scientific) according to the manufacturer's instructions. For quality control in TMT experiments, reference samples were prepared by pooling all samples, and 10 µg of reference samples were labelled with 126 and 127N TMT tags. Each TMT channel was freshly dissolved in anhydrous acetonitrile (ACN) at a ratio of 0.8:80 (w/v, mg/L). After incubation for 1 h at room temperature (23°C), the reaction was quenched by adding 3.5 µL of 5% hydroxylamine and incubated for 15 min, and the samples were combined and dried. Further, we separated total peptides into 10 fractions using basic reverse‐phase liquid chromatography, and vacuum‐dried each eluted peptide sample. For liquid chromatography‐tandem mass spectrometry (LC‐MS/MS), we diluted the fractionated peptides with mobile phase A (99.9% water with 0.1% formic acid (FA)).

### 2.10 LC‐MS/MS Analysis for Proteomics

We analysed the dissolved samples using an Orbitrap Fusion Lumos mass spectrometer (Thermo Fisher Scientific) coupled with an EASY‐nLC (Thermo Fisher Scientific) equipped with a nanoelectrospray source. Samples were trapped on a 75 µm × 2 cm C18 precolumn (nanoViper, Acclaim PepMap100, Thermo Fisher Scientific) before being separated on an analytical C18 column (75 µm × 50 cm PepMap RSLC, Thermo Fisher Scientific) for 120 min at a flow rate of 250 nL/min. Mobile phases A and B comprised 0% and 80% acetonitrile containing 0.1% formic acid, respectively. The LC gradient started with 5% B for 5 min and then 5%–7% B buffer for 5 min. It was ramped to 25% B buffer for 78 min, followed by a gradient from 25% to 40% B buffer for 13 min, 40%–95% for 5 min, and 95% for 8 min. Finally, the solution was converted to 5% buffer B for 1 min, after which it was washed for 10 min with 5% buffer B. During chromatographic separation, Orbitrap Fusion Lumos was operated in data‐dependent mode, automatically switching between MS1 and MS2. The full‐scan resolution was 120,000 and the maximum ion injection times for the full and MS/MS scans were 100. The scan range was 400–2000 m/z; we performed MS2 scans using HCD fragmentation (37.5% collision energy) and excluded previously fragmented ions after 30 s at a concentration of 10 ppm. The electrospray voltage was maintained at 2.0 kV, and the capillary temperature was set to 275°C.

### 2.11 ELISA for EV Protein Detection

We isolated and lysed the EV proteins according to the standardized protocol described above. First, tdEVs were isolated using immunoaffinity beads, and then lysed by agitation in RIPA buffer at 4°C for 30 min. Subsequently, EV lysates were centrifuged at 20,000 × *g* for 15 min at 4°C, and the protein concentration of the supernatants was measured using the Bradford assay (Bio‐Rad, Hercules, CA). We used equal amounts of EV proteins for the ELISA. All the processes were performed in accordance with the manufacturer's protocol for the ELISA kit used. Experiments for each EV protein sample were performed in duplicate, and mean values were calculated. The ELISA kits used in this study were ECM1 (Abcam, ab246524), RAB5C (MyBioSource, Vancouver, BC, Canada, MBS8806475), BTD (MyBioSource, MBS7239698), and MBL2 (Abcam, ab193709).

### 2.12 Immunohistochemistry for Detection of ECM1, RAB5C, BTD and MBL2 in Paraffin‐Embedded Tissue Sections From Breast Cancer Patients

Paraffin‐embedded tissue sections were deparaffinized and rehydrated through sequential immersion in xylene, followed by 100%, 95%, and 70% ethanol, and finally distilled water. Antigen retrieval was performed in FLEX Target Retrieval Solution High pH 9.0 (Dako, Agilent Technologies, Santa Clara, CA, USA) at 95°C for 20 min using the PT LINK system. Endogenous peroxidase activity was blocked by incubating sections in 3% hydrogen peroxide (Duksan, Ansan, South Korea) for 10 min, followed by two 5‐min washes in Tris‐buffered saline (TBS). Sections were then incubated with primary antibodies diluted in TBS at 25°C for 1 h: ECM1 (MA1‐19051, Thermo Fisher Scientific, 1:50), RAB5C (PA5‐36606, Thermo Fisher Scientific, 1:100), BTD (PA5‐28180, Thermo Fisher Scientific, 1:100), and MBL2 (A5825, Abclonal, Woburn, MA, USA, 1:100). After three 5‐min TBS washes, secondary antibodies were applied for 20 min at 25°C: Envision+ System‐HRP Anti‐Mouse (K4001, Dako) for ECM1, and Envision+ System‐HRP Anti‐Rabbit (K4003, Dako) for RAB5C, BTD, and MBL2. Sections were then washed in TBS and developed with DAB solution (K3468, Dako) for 5 min, followed by rinsing in distilled water. The sections were counterstained with haematoxylin for 10 min, washed under running tap water, dehydrated in a graded ethanol series (70%, 95%, and two changes of 100%), cleared in xylene, and mounted with coverslips.

### 2.13 Selection Criteria for the EV Protein Signature in ELISA Dataset

Logistic regression analysis was conducted to elucidate the relationship between EV proteins, treating them as independent variables. Logistic regression generates coefficients, along with corresponding standard errors and significance levels, to develop a predictive formula for the logit transformation of the probability (logit(p)) of the presence of the characteristic of interest (Peduzzi et al. 1996). In this study, the dichotomous dependent variable was represented as the predicted probability index. EV protein markers were included as independent variables in the logistic regression model. The significance level (*α*) was set to 0.05, and variables with *p* values exceeding 0.1 were excluded from the model. After that, logistic regression coefficients were computed to establish a dichotomous dependent variable for each marker's influence on the outcome. The goodness of fit of the logistic regression model was assessed using overall model fit statistics. A *p* value of less than 0.05 was considered indicative of at least one independent variable contributing significantly to the prediction of the outcome. Statistical analyses were performed using MedCalc software (v20.014; The MedCalc Software Ltd., Ostend, Belgium). The logistic regression equations were described in Table S2.

### 2.14 Receiver Operating Characteristic (ROC) and Survival Analysis

We performed ROC analysis of EV protein markers on plasma samples from patients with BC using MedCalc (version 20.014, MedCalc Software Ltd., Ostend, Belgium). We used univariate ROC analysis for each EV protein marker to obtain the ROC curve, area under the curve (AUC), AUC standard error (SE), and 95% confidence interval (CI), and evaluated the diagnostic power of the EV protein marker combinations (Table S2). After performing a univariate ROC analysis on each combination, we chose the ‘Best’ combination with the highest AUC and the lowest SE of the AUC. Cox proportional hazard regression analysis was performed to examine the correlation between protein alterations and survival. Prism (GraphPad Software, version 9.0.0; San Diego, CA, USA) was used to calculate the log‐rank *p* values, hazard ratios (HRs), and 95% CIs. Survival differences were visualized by generating Kaplan–Meier survival plots. Recurrence‐free survival refers to the time until the first recurrence event or death, and overall survival refers to the time from surgery to the last follow‐up or death.

### 2.15 Data Processing

We processed raw MS files using MaxQuant version 2.1.4 (Matrix Science, Chicago, IL, USA) with the Andromeda search engine against the UniProt human protein reference proteome database, including forward and reverse sequences and common contaminants (Cox et al. 2011; Cox and Mann 2008). To quantitatively compare the protein abundance in each sample, we calculated differentially expressed proteins (DEPs) using the reporter‐intensity‐corrected value. Low‐confidence peptides were filtered with a false discovery rate of at least 1% using a target‐decoy search strategy. To ensure fidelity, proteins identified more than twice in the three samples were considered valid. For comparative analysis, reporter intensity‐corrected values were normalized using log2 transformation. We selected DEPs using significance analysis of the volcano plot in Perseus (Tyanova et al. 2016), which was performed based on a *p* value < 0.05 and fold change > 2.

### 2.16 Hybrid Machine Learning Algorithms

All EV protein intensity data were handled through a customized ML algorithm that hybridized three individual algorithms: LSBoost for regression of result mining and convolutional neural network (CNN)‐support vector machine (SVM) for classification of clinical patients’ status. Figure S1 shows the working principle of the hybrid algorithm, which we call LSBoost‐CNN‐SVM. Initially, we entered the data consisting of the intensities of 985 proteomic biomarkers into the algorithm. The data form a large dataset by applying parallel‐learning functions based on ensemble models to the algorithm. The entire dataset was then randomly divided into a training set (70%) and a test set (30%) to avoid classification bias. From these datasets, we extracted multiple indices to identify correlations between biomarkers and adapted the indices for regression learning and converted them into characteristic equations. We used a parallel learning function to efficiently learn large‐scale data; after the training was completed, we analysed the correlations using validation indicators (root mean square error [RMSE]). The CNN‐SVM algorithm was applied to the top four biomarkers selected in ascending order of RMSE values using the LSBoost algorithm. As the input layer of the CNN‐SVM algorithm is combined with the LSBoost algorithm, the raw dataset of 985 × 130 data (number of biomarkers × number of samples) is inputted. In a CNN, learning is conducted based on specified hyperparameters; after feature extraction, these are converted into an activation map. Finally, classification learning into four classes (normal, TNBC w/o recur, TNBC with recur, and other subtypes) was completed using the SVM classifier. We showed the description of the working process by using the summarized code of the hybrid algorithm in detail in Figure S2. All experiments were performed using MATLAB R2022b (MathWorks, Inc., Natick, MA, USA) on a personal computer equipped with an Intel Core i9‐12900KS CPU operating at 3.40 GHz, 32 GB of RAM, and an NVIDIA GeForce RTX 3090. The auxiliary code of the LSBoost algorithm used in this experiment is published at https://github.com/SeongminHA/Machine‐learning‐reinforced‐proteomic‐profiling‐of‐circulating‐extracellular‐vesicles‐in‐et‐al.

## 3 Results

### 3.1 Quantitative Proteomics Analysis and Diagnostic Signature of tdEVs

Proteomic analysis of tdEVs isolated from the plasma of 30 healthy individuals and 100 patients with BC using the microfluidic system identified 3,171 proteins (Supplementary Data). To ensure the reliability and accuracy of tdEV isolation, both qualitative and quantitative characterizations were performed in adherence to the MISEV guidelines, as described in our previous studies (Gwak et al. 2021; Kim et al. 2021). NTA revealed a unimodal size distribution ranging from 50 to 300 nm, consistent with the typical size of EVs, and SEM imaging confirmed the presence of spherical vesicles with characteristic tdEV morphology, further validating their identity (Figure S3). Additionally, flow cytometry validated the presence of EV surface markers, including CD9, CD63, and CD81, on tdEVs attached to singlet beads (Figure S4A). Notably, flow cytometry analysis demonstrated the presence of EV surface markers across all groups, irrespective of disease status (Figure S4B,C). However, compared to the healthy control group, the cancer groups exhibited generally higher levels of EV surface marker expression, likely reflecting the enrichment of tdEVs achieved through the microfluidic system. Collectively, these results underscore the successful isolation and comprehensive characterization of tdEVs, highlighting the reliability and robustness of our microfluidic platform.

**FIGURE 2:**
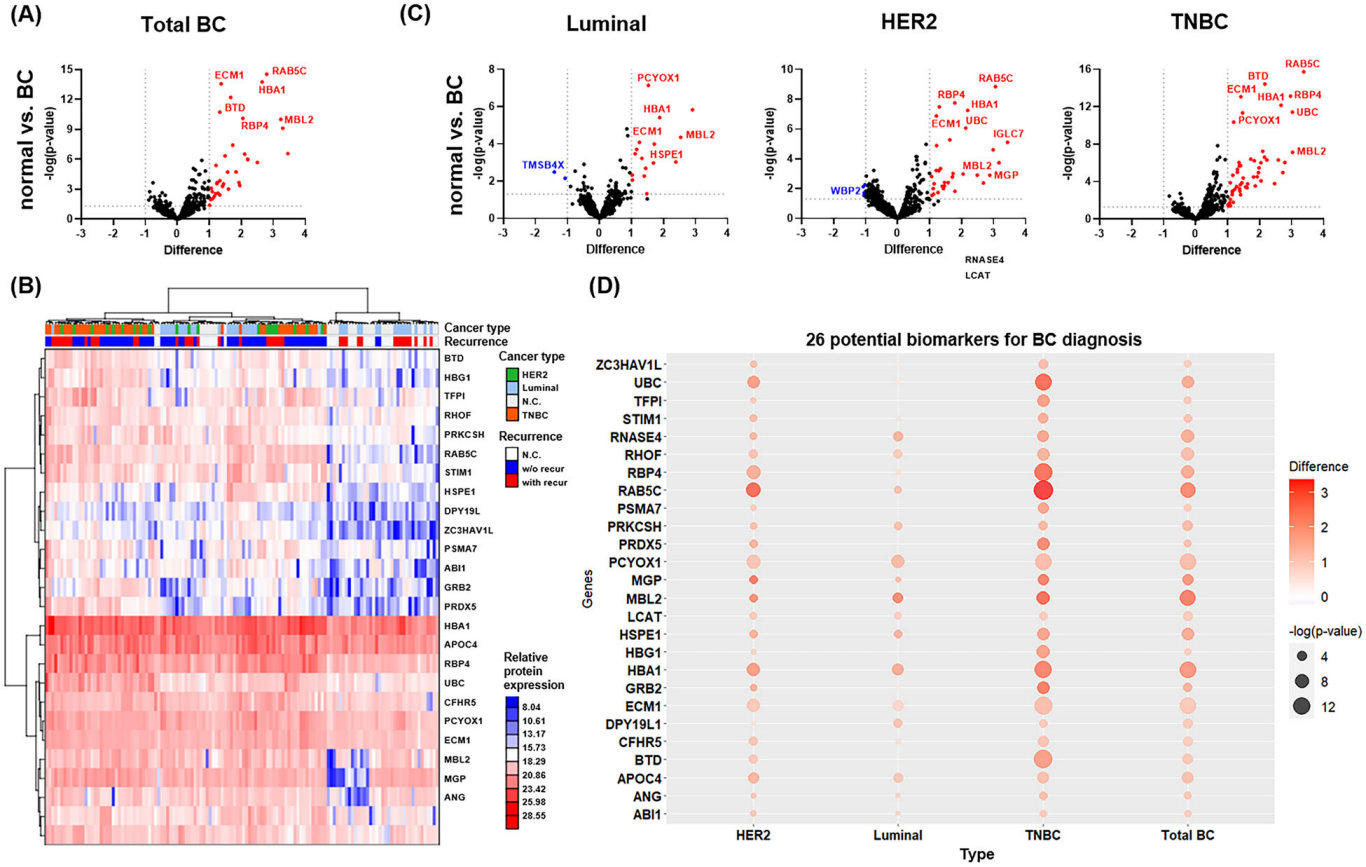
Comparative analysis of 985 tdEV proteins by BC subtype. (A) The volcano plot analysis identified DEPs for breast cancer for 985 tdEV proteins from healthy people (*n* = 30) and total breast cancer patients (*n* = 100) (red dot: *p* < 0.05, log2(difference) > 1). (B) Heatmap of Euclidean distances and associated hierarchical clustering dendrograms between 26 significant proteins and patients. In this panel, individual entities are labelled with two colour codes. The first colour represents the breast cancer subtype (HER2 is green, luminal is light blue, N.C. is grey, and TNBC is orange). The second colour codes the recurrence of cancer patients (white for N.C., blue for without recurrence of cancer, and red for with recurrence of cancer. (C) The volcano plot analysis identified DEPs for breast cancer from among 985 tdEV proteins from healthy individuals and each subtype (luminal, HER2, and TNBC) of breast cancer patients (red dot: *p* < 0.05, log2(difference) > 1), blue dot: *p* < 0.05, log 2(difference)←1). (D) Bubble plot analysis showed multiple significant differences by subtype for the 26 possible biomarkers identified in the volcano plot showing differences between breast‐cancer patients and healthy people. BC, breast cancer; DEPs, differentially expressed proteins; N.C., normal control; tdEV, tumour‐derived extracellular vesicles; TNBC, triple‐negative breast cancer.

The expression of proteins reported by Kugeratsky et al. as exosomal markers was validated in LC‐MS/MS analyses (Kugeratski et al. 2021). For non‐exosomal markers (exclusion markers, Figure S5 in blue text), with the exception of CANX, most proteins were either not identified (e.g., HMGB1, NOCL1, HMGB2, HMGB3, SLIRP) or had relatively low expression levels compared to exosomal markers (Figure S5 in red text). Furthermore, among the 3171 EV proteome entries identified, Gene Ontology analysis of cellular components revealed ‘extracellular exosome’ to be the most relevant term, with a majority of the top 10 relevant components being extracellular region or membrane‐associated proteins (Table S3). Of the 3171 proteins identified, 2458 proteins were identified in at least 10 individuals in a group (Figure S6A). To compare the proteomes of normal and various BC patients more accurately, we performed a comparative analysis using the 985 proteins identified in more than 70% of all groups. We found that the HER2 type and TNBC could be clearly distinguished from normal individuals, while the luminal type was somewhat ambiguous (Figure S6B,C). When the 985 proteins were compared, it was difficult to distinguish between patients with and without recurrence for each subtype. However, there were some differences when subtype‐specific significant proteins were compared (Figure S7). An essential observation in cancer diagnostic studies is the selectivity between patients with tumours and healthy controls. To identify such selective biomarkers, we identified the differential expression patterns of protein markers between tumour patients and healthy controls using volcano plot analysis. After comparing the 985 proteins via the volcano plot, 26 proteins showed selective significance (*p* < 0.05, fold change > 2) between patients with tumours and healthy controls (Figure 2A). However, as shown in the hierarchical heatmap and principal component analysis plot analysis of the 26 significant proteins, it was difficult to accurately distinguish between patients with tumours and healthy controls (Figure 2B). We performed sensitivity and specificity analyses of the 5 proteins with the highest *p* values among the 26 significant proteins to distinguish between healthy controls and patients with BC (Figure S8). Notably, we found that the HBA1 protein had a higher *p* value but a lower AUC, compared with ECM1, whereas MBL2 had a higher *p* value but a lower AUC, compared with RAB5C. These data suggest that owing to the molecular heterogeneity in BC subgroups, attempts to identify biomarkers relevant to cancer diagnosis without considering these subtypes by performing whole‐BC analysis are likely to be inefficient. Therefore, we identified proteins in the BC subgroup that differed from those in healthy individuals. By analysing the volcano plot, we found 14 proteins with significantly high expression and 2 proteins with significantly low expression in the healthy and luminal groups, 27 proteins with significantly high expression and 5 proteins with significantly low expression in the HER2 group, and 47 proteins with significantly high expression in the TNBC group (*p* < 0.05, fold change > 2) (Figure 2C). To investigate whether protein comparisons between each BC subgroup and healthy controls showed similar patterns, we examined the significant protein expression changes between patients with tumours and healthy controls using bubble plots for the 26 proteins that showed selective significance. The results showed that some proteins showed similar patterns in protein comparisons between each BC subgroup and healthy controls, whereas others showed different patterns in each group (Figure 2D). Information on each protein can be found in Table 1 and the Supplementary Data in the Excel file.

**TABLE 1:** Twenty‐six significant proteins to distinguish between healthy controls and patients with breast cancer.

Gene names	UniProt accessions	Protein names	*p* value	Fold change
*PCYOX1*	Q9UHG3	Prenylcysteine oxidase 1	4.84E‐12	2.749
*HBA1*	P69905	Haemoglobin subunit alpha	5.43E‐12	4.846
*ECM1*	Q16610	Extracellular matrix protein 1	5.45E‐12	2.238
*MBL2*	P11226	Mannose‐binding protein C	1.21E‐10	6.665
*RAB5C*	P51148	Ras‐related protein Rab‐5C	4.33E‐10	5.913
*RHOF*	Q9HBH0	Rho‐related GTP‐binding protein RhoF	5.38E‐08	2.673
*RNASE4*	P34096	Ribonuclease 4	7.16E‐08	3.486
*RBP4*	Q5VY30	Retinol‐binding protein 4	1.07E‐07	3.614
*HSPE1*	P61604	10 kDa heat shock protein, mitochondrial	7.50E‐07	3.593
*UBC*	P0CG48	Polyubiquitin‐C	7.93E‐07	3.701
*APOC4*	P55056	Apolipoprotein C‐IV	1.39E‐06	2.617
*MGP*	P08493	Matrix Gla protein	5.45E‐06	4.852
*PRKCSH*	K7ELL7	Glucosidase 2 subunit beta	2.64E‐05	2.849
*BTD*	P43251	Biotinidase	6.20E‐05	2.337
*LCAT*	P04180	Phosphatidylcholine‐sterol acyltransferase	1.32E‐04	2.146
*CFHR5*	Q9BXR6	Complement factor H‐related protein 5	1.40E‐04	2.099
*DPY19L1*	A0A1B0GW05	Probable C‐mannosyltransferase DPY19L1	1.41E‐04	2.165
*GRB2*	P62993	Growth factor receptor‐bound protein 2	2.82E‐04	3.229
*STIM1*	Q13586	Stromal interaction molecule 1	9.88E‐04	2.452
*ANG*	P03950	Angiogenin	2.58E‐03	2.327
*PSMA7*	O14818	Proteasome subunit alpha type‐7	3.48E‐03	2.146
*TFPI*	P10646	Tissue factor pathway inhibitor	3.75E‐03	2.253
*PRDX5*	P30044	Peroxiredoxin‐5, mitochondrial	3.80E‐03	2.545
*ZC3HAV1L*	Q96H79	Zinc finger CCCH‐type antiviral protein 1‐like	4.43E‐03	2.088
*ABI1*	A0A0A0MRT6	Abl interactor 1	7.33E‐03	2.111
*HBG1*	A0A2R8Y7 × 9	Haemoglobin subunit gamma‐1	9.76E‐03	2.067

**FIGURE 3:**
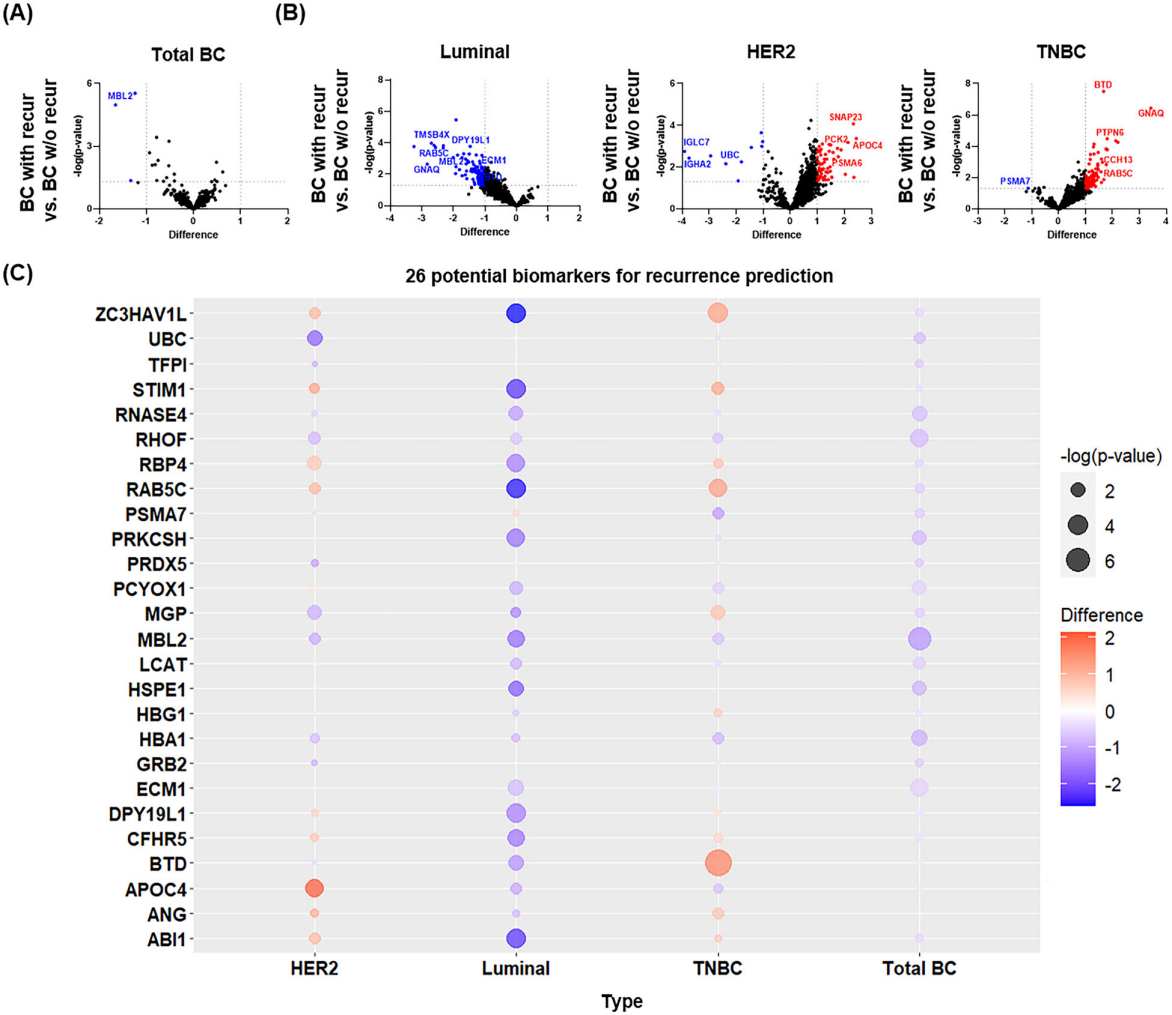
Comparative analysis of 985 tdEV proteins in patients with and without recurrence by BC subtype. (A) The volcano plot analysis identified DEPs from among 985 tdEV proteins from breast cancer patients with (*n* = 37) and without recurrence (*n* = 63) from the total cohort of breast cancer patients (*n* = 100) (blue dot: *p* < 0.05, log 2(difference)←1). (B) DEPs identified by volcano plot analysis of 985 tdEV proteins from breast cancer patients with and without recurrence for each subtype (red dot: *p* < 0.05, log 2(difference) > 1), blue dot: *p* < 0.05, log 2(difference)←1). (C) Bubble plot analysis showed multiple significant differences by recurrence and subtype for the 26 possible biomarkers identified in the volcano plot showing differences between breast cancer and healthy people.BC, breast cancer; DEPs, differentially expressed proteins; tdEV, extracellular vesicles.

To identify predictive markers for early recurrence among patients with BC, we performed a proteomic comparison of all patients with breast cancer and subtypes. We found that MBL2 protein was downregulated in recurrent patients compared with that in non‐recurrent patients (Figure 3A). When analysed by each BC subtype, the luminal subtype showed statistically significant downregulation of some protein markers compared with non‐recurrent patients. Conversely, in more aggressive phenotypes characterized by unfavourable prognoses, such as HER2 and TNBC, most proteins were predominantly upregulated in recurrent patients (Figure 3B). RAB5C protein was decreased in recurrent patients but only in the luminal type and increased in HER2 and TNBC. We also found subtype‐specific differences in recurrent patients for each of the aforementioned proteins with significance in the 26 differentially expressed proteins with BC (Figure 3C), which, similar to previous data, suggests potential subtype‐specific molecular pathogenesis affecting prognosis and recurrence in BC subgroups. Fiorenza et al. reported that existing analytical methodologies are limited in depicting real‐world patients because they do not consider the complex molecular landscape of subtype differences or individual prognoses (De Rose et al. 2022). Owing to this molecular heterogeneity, attempts to perform whole‐BC analyses to identify appropriate recurrence‐related biomarkers without considering these subtypes are likely to be ineffective. To overcome this limitation, we applied ML techniques optimized for large‐scale data investigation, with a particular focus on TNBC, which has a significantly worse prognosis at recurrence.

### 3.2 Training of the Hybrid Machine Learning Algorithms

To achieve a high classification accuracy in multiclass datasets, characteristic equations that can be used to interpret large datasets and complex data structures are required. Applying basic CNNs to multiclass datasets can result in overfitting, which reduces learning reliability and increases data bias. Therefore, before classifying the classes, we applied a hybrid‐type least‐squares boosting (LSboost) algorithm that combines the advantages of the AdaBoost and gradient boost models to a dataset of 985 identified proteomic biomarker intensities. This algorithm automatically extracts characteristic equations suitable for data analysis and assigns weights in a manner that minimizes the mean‐square error of the difference between the actual and predicted values during the regression analysis. The learning results of the dataset applied to the algorithm were compared with 985 existing raw proteomic datasets to calculate the RMSE values using the following equation:

(1) $$RMSE=\sqrt{\frac{1}{n}\sum_{i=1}^{n}{\left(\hat{y_{i}}-y_{i}\right)}^{2}}$$

where *n* is the number of observations, $\hat{y_{i}}$ is the actual value of the *i*th observation, and $y_{i}$ is the predicted value of the *i*th observation.

**FIGURE 4:**
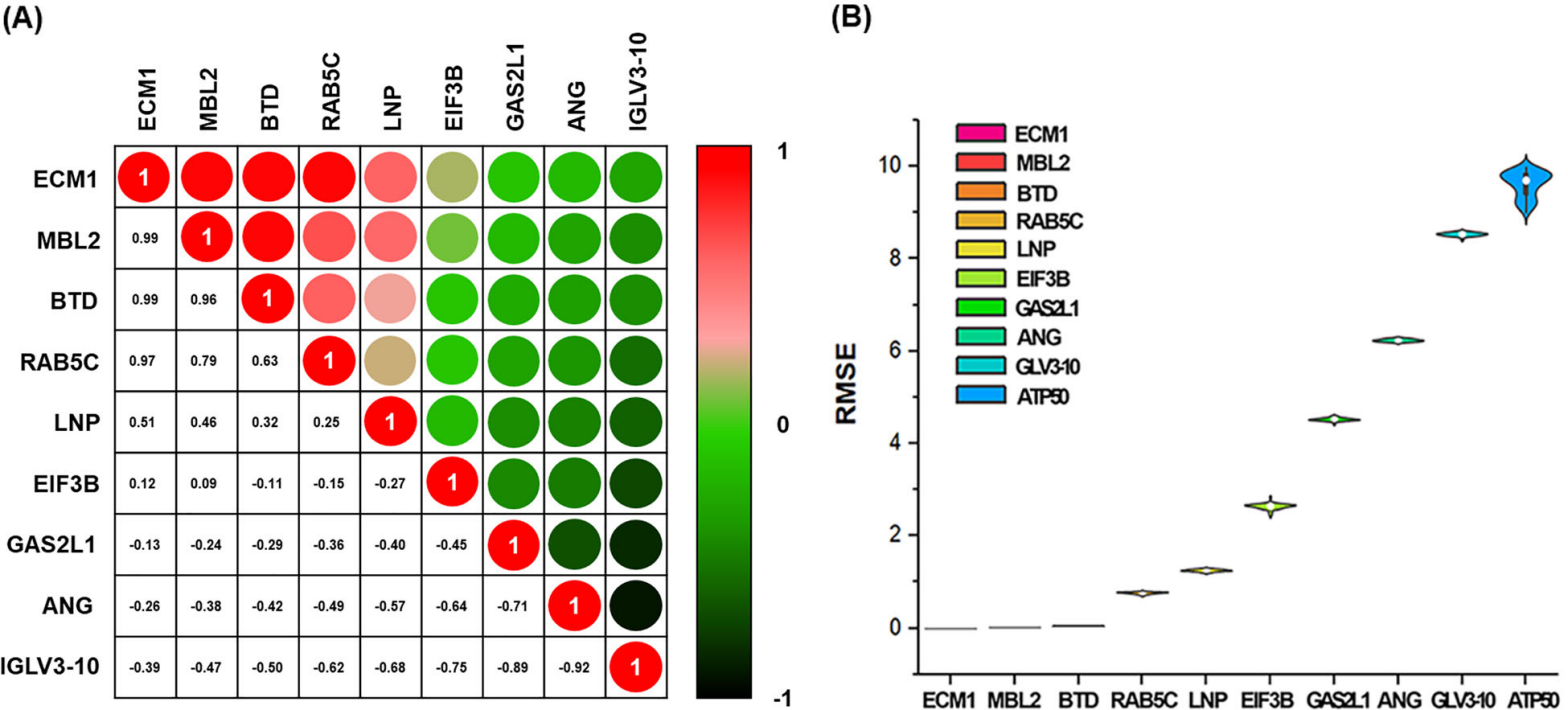
Correlation matrix according to regression learning of biomarker intensity using the LSBoost algorithm. The top 10 biomarkers are presented in ascending order of normalized RMSE (Root Mean Squared Error) values using the algorithm's characteristic equation for the 985 biomarkers extracted through proteomics analysis. (A) The correlation matrix shows the relationship between each biomarker among the top 10 biomarkers. (B) The plot of the results of 50 iterations of the algorithm as the distribution of the RMSE across biomarkers.

Figure 4A shows the correlation between each biomarker by selecting the top 10 biomarkers in ascending order among the normalized RMSE values of 985 biomarkers extracted using the characteristic equation of the LSBoost algorithm. The correlation coefficients of normalized RMSE values between the four biomarkers (ECM1, MBL2, BTD, and RAB5C) had over 0.5. The closer the correlation coefficient is to 1, the stronger the relationship between biomarkers, indicating that the model fits the data well and provides more accurate predictions. Additionally, the small residuals of the top 4 biomarkers showed superiority over those of the other biomarkers (Figure 4B), according to the plot of the results of 50 iterations of the LSBoost algorithm as the distribution of the RMSE across biomarkers.

### 3.3 Selection of the Potential tdEV Protein Biomarkers Using the LSBoost‐CNN‐SVM Algorithm

**FIGURE 5:**
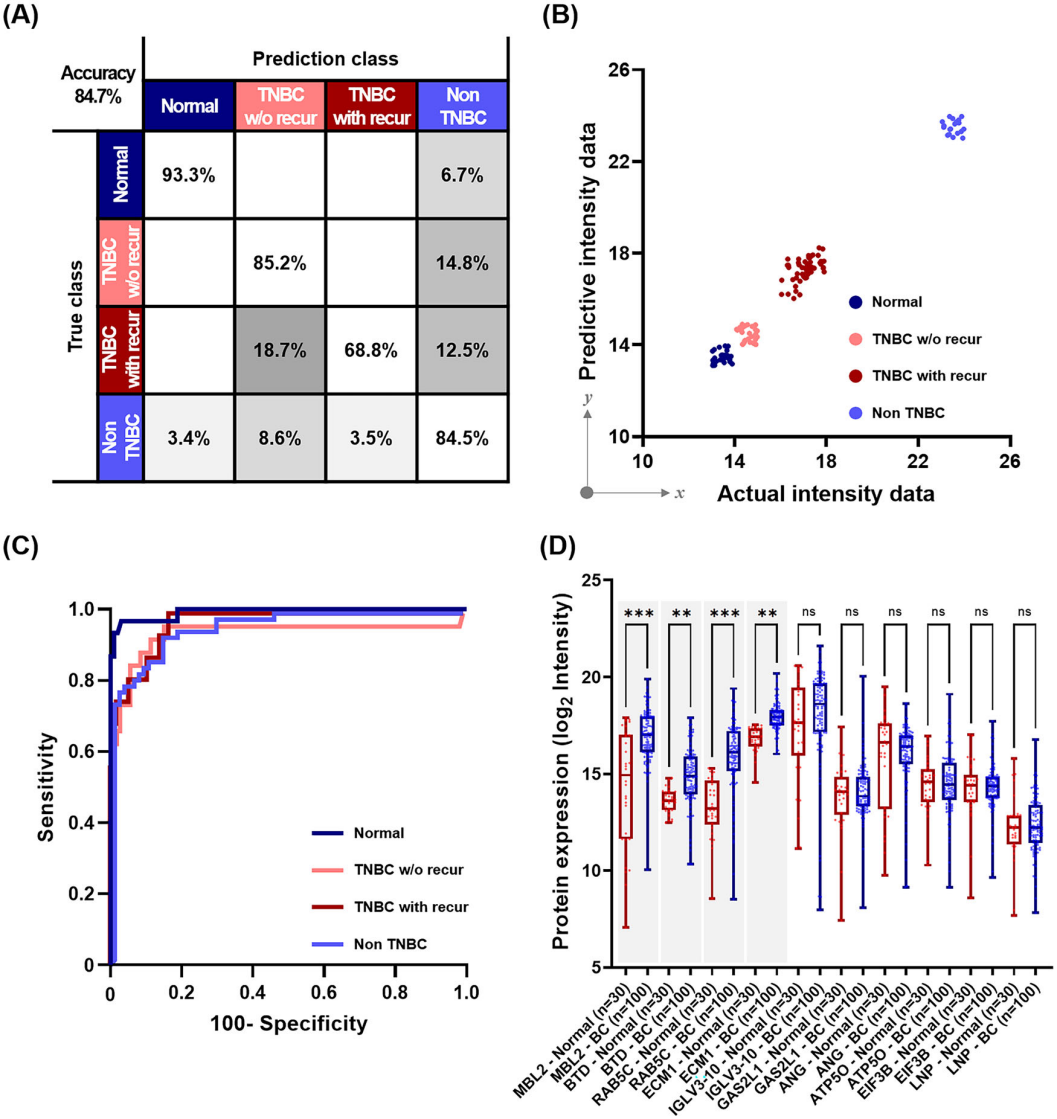
Training results of the hybrid algorithm (LsBoost‐CNN‐SVM). (A) Confusion matrix of training results for four classes (Normal, TNBC w/o recur, TNBC with recur, and non‐TNBC). (B) Scatter plot of actual and predicted data in the training set. (C) ROC curve and AUC values of each class. (D) Protein expression levels of the top 10 predicted proteins as significant in differentiating patients with BC from normal. Statistical analyses were performed using an unpaired Student's t‐test between two groups.**p < 0.01; ***p < 0.001. AUC, area under the ROC curve; ns, not significant; ROC, receiver operating characteristic; TNBC, triple‐negative breast cancer; with recur, with recurrence; w/o recur, without recurrence.

We applied 985 biomarkers weighted using the LSBoost algorithm to the CNN‐SVM algorithm, which combines a CNN model specialized for large‐scale data classification and an SVM as a multi‐classifier. Among the biomarkers weighted from the LSBoost algorithm, 10 biomarkers in ascending order of RSME are combined and passed through the kernel filter to find the best combination of biomarkers for four classifications: normal, TNBC w/o recur, TNBC with recur, and other subtypes. The confusion matrix for the training results is shown in Figure 5A. The true positive rate (TPR) and false negative rate (FNR) were evaluated for four classifications with an average accuracy of 84.7%. Notably, the developed LSBoost‐CNN‐SVM algorithm exhibited an accurate classification performance in TNBC w/o recur class, with 85.2% sensitivity and 90.9% specificity. Figure 5B shows a scatter plot of the training results, which were used to evaluate the correlation linearity between the actual and predicted data. Actual intensity data on the *x*‐axis refers to the normalized combination intensity of the actual quantified four biomarkers, and predictive intensity data on the *y*‐axis refers to the normalized combination intensity of the four biomarkers predicted from the training results of the algorithm. The results suggest that there is a high correlation between actual patient data and prediction data for each type of breast cancer using a combination of four biomarkers determined through a hybrid algorithm. Each colour‐coded class forms a cluster, indicating the high accuracy of the model. Based on these results, we plotted the ROC curve, which shows the prediction performance, including sensitivity and specificity, of the LSBoost‐CNN‐SVM algorithm. The AUC of the ROC curve represents the performance reliability, and the AUC values were greater than 0.94 in every class (Normal, 0.99; TNBC w/o recur, 0.94; TNBC with recur, 0.97; and other subtypes, 0.95), which was close to 1.0, indicating that the performance of the algorithm was outstanding (Figure 5C) (Mandrekar 2010). Further, we analysed the expression levels of the predicted proteins from the data analysed by LC‐MS/MS in normal individuals and patients with BC. Consequently, we found the top 10 predicted proteins identified by the LSBoost‐CNN‐SVM algorithm. Among them, four EV proteins (ECM1, MBL2, BTD, and RAB5C) were confirmed as significant in differentiating patients with BC from normal individuals (Figure 5D). We evaluated the classification accuracy of each EV protein marker alone or in combination, based on the results of training the CNN‐SVM algorithm with weighted outputs for single and multiple EV protein markers using the LSBoost algorithm (Table S4). High AUC values > 0.9 were shown across all classes when three or more protein markers were combined.

### 3.4 Evaluation of Multi‐Marker Combinations

To enhance the performance of the selected EV protein markers identified by the LSBoost‐CNN‐SVM algorithm in differentiating patients with BC and normal individuals, the optimal combination was evaluated using LC‐MS/MS data. A signature combining ECM1, MBL2, BTD, and RAB5C (‘tdEV_protein_ score’) achieved the best performance in BC diagnosis; therefore, it was selected for subsequent clinical evaluation (Table S5). Then, we explored the potential of tdEV_protein_ score as a predictor of clinical, histological, and demographic features in our study cohort. As depicted in Table 2, we identified the correlation of the tdEV_protein_ score with patients’ demographical information, such as gender, ethnicity, and age, in the mass spectrometry study (*N* = 130). Given that all participants in our study were female and of East Asian ethnicity, we did not consider gender and race in our analysis. Our findings showed no significance between age distributions in patients with BC (*p* = 0.5812). In addition, when examining age‐related correlations within the normal control group, we found no significant differences. The average age was 49.3 ± 7.8 years (mean ± standard deviation) for healthy participants, closely aligning with 51.9 ± 10.8 years for the BC patient group (Figure S9). Subsequently, the correlations with clinical and histopathological characteristics of BC patients were evaluated. For instance, clinical characteristics, such as subtype (*p* = 0.04), metastasis (*p* < 0.001), and recurrence (*p* < 0.001) were strongly correlated with the tdEV_protein_ score. Upon assessing the association with early tumour incidence, we found that patients experiencing early recurrence had elevated EV_protein_ scores. This observation aligns with findings by Pedersen et al., who noted that breast cancer patients with early recurrence faced poorer outcomes compared to those with late recurrence (Pedersen et al. 2022). On the other hand, factors such as cancer stage, tumour size, lymph node involvement, Ki‐67 level, CA15‐3 and CEA levels, and treatment approach did not exhibit significant correlations. Compared to conventional diagnostic methods like CA15‐3, CEA, and mammography, our approach has higher sensitivity (Figure S10). These findings suggested the tdEV_protein_ score's potential as a predictive tool for patient recurrence, prognosis, and the management of aggressive TNBC.

**TABLE 2:** Clinical characteristics of the enrolled BC patients in the proteomic analysis set.

Variables	Patients (*N* = 100)	Mean EV_protein_ score	95% CI	*p* value
**Age**				
≤ 30's	14	0.909	0.816–1.003	0.5812
40's	34	0.831	0.744–0.917	
50's	29	0.879	0.808–0.855	
60's	18	0.913	0.855–0.971	
≥ 70's	5	0.934	0.875–0.993	
**TNM Stage**				
0–I	38	0.802	0.790–0.934	0.577
II	46	0.908	0.868–0.948	
III–IV	12	0.889	0.815–0.964	
NA	4	0.592	−0.128–1.312	
**Subtype**				
Luminal	33	0.777	0.677–0.876	**0.04**
HER‐2/neu	24	0.911	0.850–0.972	
TNBC	43	0.930	0.904–0.956	
**Tumour size**				
≤ 2 cm	52	0.859	0.800–0.918	0.59
> 2 cm, ≤ 5 cm	39	0.899	0.852–0.947	
> 5 cm	5	0.944	0.879–1.010	
Unknown	4			
**Lymph node invasion**				
None	64	0.878	0.829–0.928	0.8
1–3 lymph node	25	0.865	0.776–0.955	
≥ 4 lymph nodes	8	0.874	0.758–0.990	
Unknown	3			
**Metastasis**				
No	64	0.849	0.795–0.903	**< 0.001**
Yes	33	0.909	0.865–0.953	
Unknown	3			
**Ki‐67**				
Low (< 15)	28	0.812	0.711–0.913	0.36
High (≥ 15)	62	0.907	0.871–0.943	
Unknown	10			
**CA15‐3 (U/mL)**				
Low (< 25)	87	0.869	0.826–0.912	0.63
High (≥ 25)	10	0.906	0.825–0.987	
Unknown	3			
**CEA (ng/mL)**				
Low (< 5)	90	0.869	0.827–0.912	0.98
High (≥ 5)	8	0.922	0.847–0.996	
Unknown	2			
**Recurrence**				
No	63	0.754	0.629–0.878	**< 0.001**
Yes (before 2 years)	14	0.938	0.910–0.966	
Yes (within 2–5 years)	23	0.812	0.708–0.916	
**Regimen**				
ACT‐based chemotherapy	63	0.893	0.848–0.938	0.33
Antibody‐based targeted therapy	14	0.900	0.807–0.992	
Others (platin, fluorouracil, etc.)	16	0.866	0.774–0.957	
Unknown	7			

Abbreviations: ACT, adriamycin‐cytoxan‐taxane; CA15‐3, cancer antigen 15‐3; CEA, carcinoembryonic antigen. Values shown in bold represent statistically significant findings.

### 3.5 Diagnostic Potential of EV Protein Marker Combination in TNBC Subtype

**FIGURE 6:**
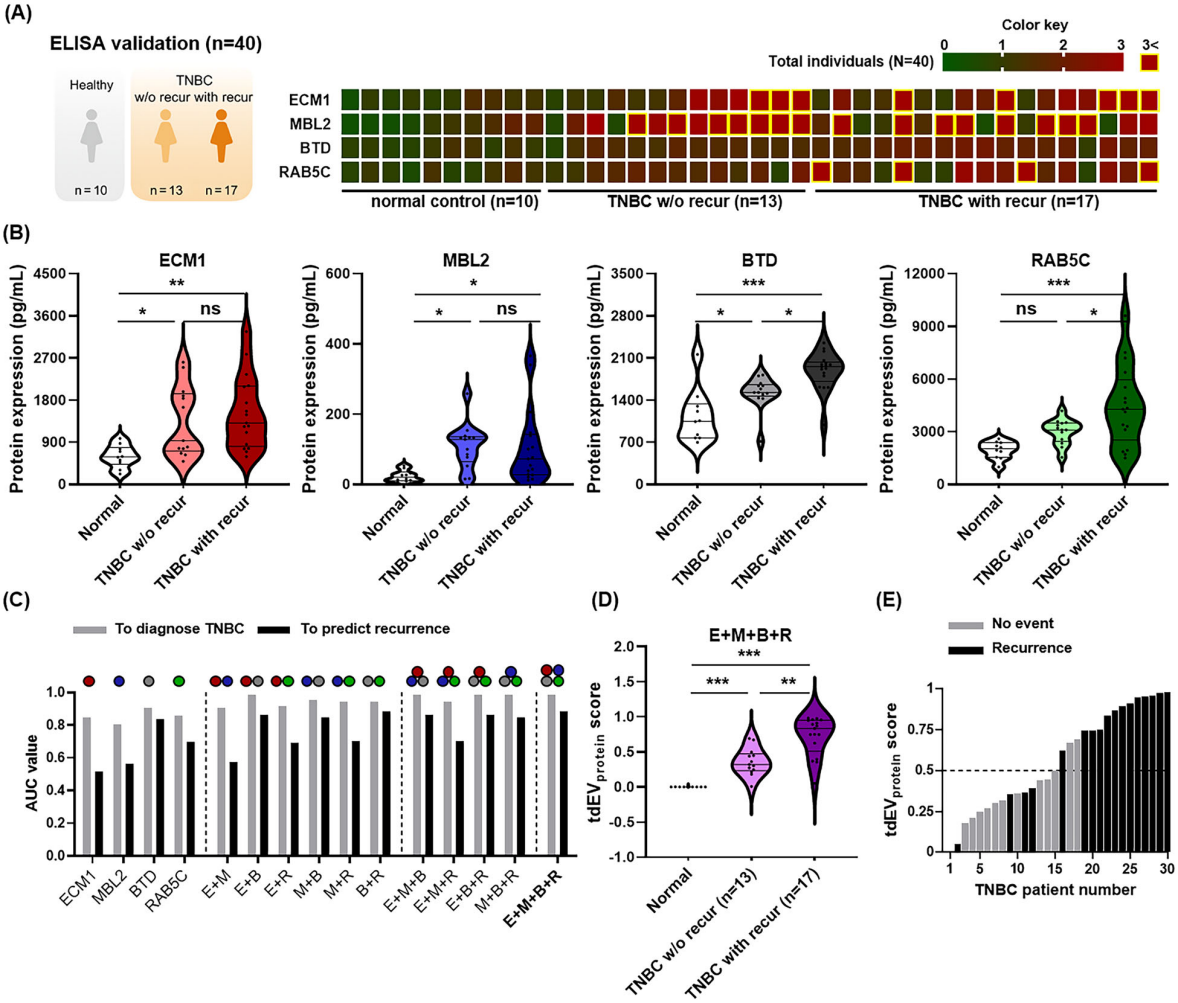
Validation of the top 4 tdEV protein markers after ML analysis using ELISA (*N* = 40). (A) Heatmap illustrating the enrichment of tdEV protein markers from TNBC w/o recur (*n* = 13) and TNBC with recur (*n* = 17) compared with normal controls (*n* = 10). The colour key represents the fold change arbitrarily standardized to the average of the normal controls. (B) Comparison of the protein expression levels of tdEV protein markers in each group, including normal controls, TNBC w/o recur, and TNBC with recur. Statistical analyses were performed using one‐way ANOVA with Turkey's multiple comparisons between three groups (ns, not significant; *p < 0.05; **p < 0.01; ***p < 0.001). (C) ROC curve analyses for single and combined EV protein markers. The AUC values were calculated using the Wilcoxon/Mann–Whitney test. (D) Comparison of the tdEV_protein_ scores between the groups. (E) The tdEV_protein_ score measuring for 30 TNBC patients. Black bars indicate the patients who underwent recurrence and grey bars indicate those with no recurrence event. AUC, area under the ROC curve; ROC, receiver operating characteristic; tdEV, tumour‐derived extracellular vesicles; TNBC, triple‐negative breast cancer; with recur, with recurrence; w/o recur, without recurrence.

Next, we conducted a comparative analysis, particularly in the aggressive subtype of TNBC. We assessed the diagnostic performance of the tdEV_protein_ score in distinguishing between TNBC patients with recurrence (TNBC with recur), TNBC patients without recurrence (TNBC w/o recur), and normal individuals. Given the clinical advantages of ELISA, such as cost‐effectiveness and rapid processing times, our study aimed to determine its potential as an alternative to the more complex and costly LC‐MS/MS. In the ELISA study, we employed heat map analysis of the data from 40 individuals to visualize the comprehensive expression patterns of the selected EV protein markers (Figure 6A). Owing to the considerable variation in the blood concentrations of individual EV protein markers across patient populations, we standardized the concentration ranges of each biomarker. This standardization process aimed to establish a normalization score for each EV protein marker by calculating the ratio of the measured concentration relative to the median concentration for each biomarker in normal controls, which enabled us to effectively compare and analyse the protein concentration levels across the test groups. For instance, among the TNBC w/o recur group, 53.8% exhibited scores more than three times higher for at least one EV protein marker. In contrast, TNBC with recur showed a more distinct pattern. A significant majority (76.5%) of patients displayed notably higher scores, and this subgroup exhibited a broader range of scores than the TNBC w/o recur group. Remarkably, the high expression levels of ECM1, MBL2, and BTD were significantly effective (*p* < 0.05) in differentiating normal samples from those of TNBC patients, as determined by analysing the absolute concentrations of these EV protein markers in plasma samples, thus highlighting their strong diagnostic potential for TNBC, independent of recurrence (Figure 6B). Specifically, BTD, in particular, was identified as the most robust indicator for TNBC, followed by RAB5C, MBL2, and ECM1. When focusing on recurrence, BTD and RAB5C emerged as significant markers (*p* < 0.05). ROC analysis further supported the excellent discriminatory ability of ECM1, MBL2, BTD, and RAB5C for TNBC diagnosis and recurrence (Figure S11). The outcomes were consistent across both proteomic and ELISA analyses, with no notable differences in their performance (Figure S12). In summary, at a clinically valuable specificity of 95%, the tdEV_protein_ score from the proteomic analysis exhibited high diagnostic precision for BC, reaching a sensitivity of 73%. For TNBC specifically, the method achieved an impressive sensitivity of 87%. An ELISA validation dataset, derived from an alternate source, confirmed these results by showing a sensitivity of 85%, thus validating the significant clinical potential of the EV protein markers identified by our research (Table 3).

**TABLE 3:** Estimated sensitivity at fixed specificity.

	Proteomic analysis			ELISA validation
	Total BC (*n* = 100) versus Control (*n* = 30)	TNBC (*n* = 43) versus Control (*n* = 30)	Non‐TNBC (*n* = 57) versus Control (*n* = 30)	TNBC (*n* = 30) versus Control (*n* = 10)
**Specificity** **(%, 95% CI)**	93.33 (77.9 to 99.2)	100.00 (88.4 to 100.0)	90.00 (73.5–97.9)	90.00 (55.5 to 99.7)
**Sensitivity** **(%, 95% CI)**	80.00 (70.8 to 87.3)	84.78 (71.1 to 93.7)	75.93 (62.4–86.5)	100.00 (88.4 to 100)
**AUC** **(95% CI)**	0.924 (0.864 to 0.963)	0.973 (0.907 to 0.997)	0.882 (0.793 to 0.942)	0.986 (0.893 to 0.998)
**Fixed 95% Specificity**	95.00%	95.00%	95.00%	95.00%
**Sensitivity** **(%, 95% CI)**	73.00 (56.00 to 83.00)	86.96 (73.91 to 95.65)	61.11 (38.89 to 74.07)	90.00 (73.33 to 100.00)

### 3.6 Prognostic Potential of EV Protein Biomarkers

In the single‐biomarker analysis, all markers exhibited remarkable AUC values, surpassing 0.75, establishing them as robust discriminators for TNBC diagnosis. BTD emerged as the most robust discriminator for TNBC diagnosis, followed by RAB5C, MBL2, and ECM1. Importantly, although using multiple markers elevated the AUC values overall, the selection of an appropriate marker combination had a more significant impact on performance (Figure 6C). For instance, combining ECM1, MBL2, BTD, and RAB5C (tdEV_protein_ score) achieved the enhanced significance in diagnosing TNBC and predicting their recurrence (Figure 6D). This approach suggests that the tdEV_protein_ score can be used to predict patient recurrence and prognosis. To explore this possibility, we conducted a comparative analysis of the tdEV_protein_ score of 30 TNBC patients from our ELISA study to validate its potential for prognosis prediction further. We established that a tdEV_protein_ score above 0.5 potentially indicates a heightened risk of recurrence (Figure 6E). Nevertheless, exceptions were noted in patients numbered 2, 9, 11, 12, 17 and 18. Among them, patients 2, 9, 11 and 12 exhibited false‐negative results. A thorough review of their clinical records revealed that these cases of false negatives were associated with the recurrence pattern. An analysis comparing scores relative to the recurrence site indicated that patients experiencing loco‐regional recurrence presented somewhat lower tdEV_protein_ scores (*p* < 0.01) compared to those with distant recurrence, supporting our hypothesis for the observed false negatives (Figure S13). Patients 17 and 18 exhibited false‐positive results. Both patients were confirmed to have died even though there were no cases of recurrence, implying that patients with higher tdEV_protein_ scores are more likely to have a worse prognosis.

**FIGURE 7:**
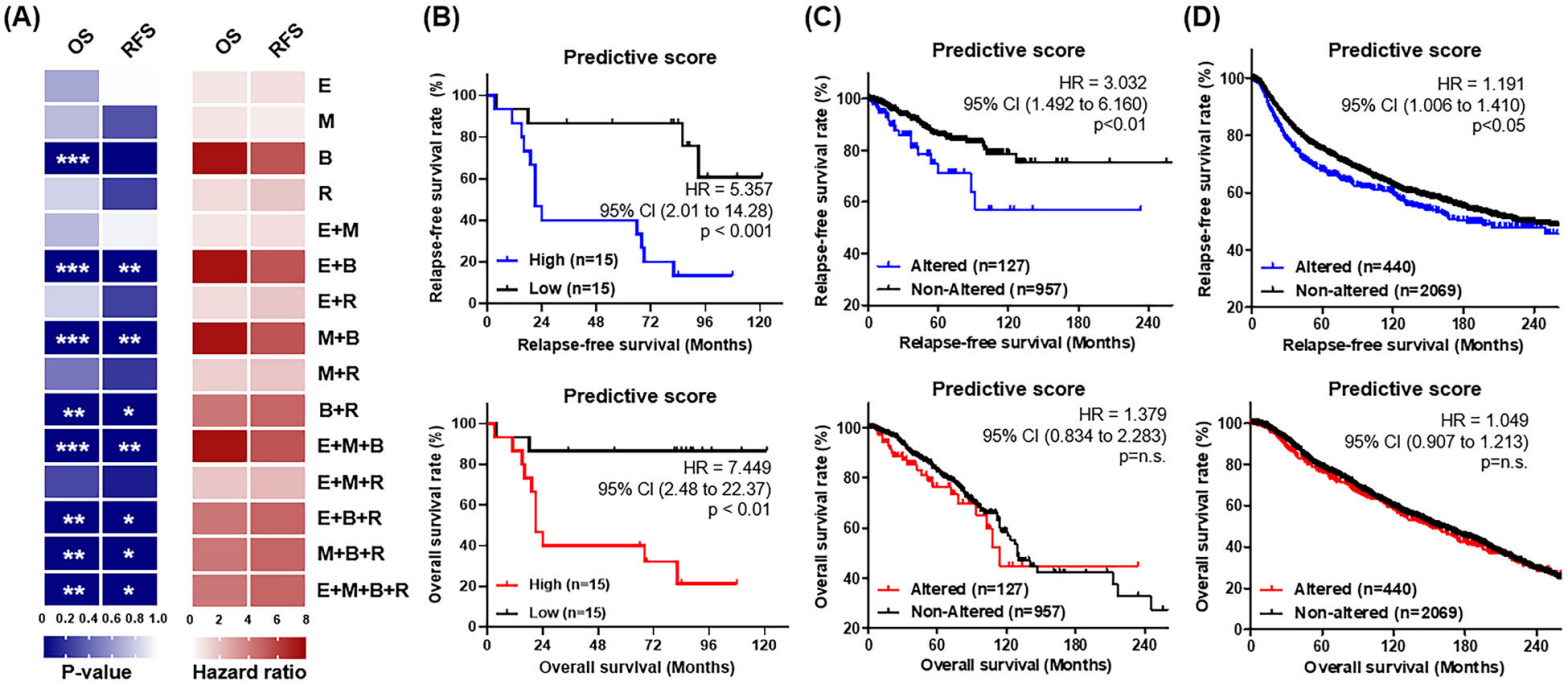
Evaluation of prognostic potential for tdEV protein markers. (A) Integrative analysis of the hazard ratio (red heatmap) and *p* value (blue heatmap) of OS and RFS tested with single and combined EV protein markers. Kaplan–Meier survival curve of RFS and OS stratified by four protein‐altered and non‐altered groups from our TNBC patient study (B), from the Pan‐Cancer Atlas (C), and METABRIC data (D). The log‐rank (Mantel–Cox) test statistic compares estimates of the HR between four protein marker high and low expression groups (*p < 0.05; **p < 0.01; ***p < 0.001). TNBC, triple negative breast cancer; EV, extracellular vesicle; HR, hazard ratio; CI, confidence interval; RFS, relapse‐free survival; OS, overall survival.

In our validation set, we examined the expression levels of ECM1, MBL2, BTD, and RAB5C, focusing on their association with patient survival outcomes. Despite the observation that other combinations of markers (ECM1 + BTD, MBL2 + BTD, and ECM1 + MBL2 + BTD) presented higher *p* values, the tdEV_protein_ signature, encompassing all four markers, was still found to be statistically significant. This signature demonstrated notable hazard ratios (HR) indicative of its relevance to both relapse‐free survival (RFS; HR = 4.821, *p* < 0.05) and overall survival (OS; HR = 4.254, *p* < 0.01), emphasizing the potential utility of this biomarker panel in predicting survival outcomes for patients within the cohort (Figure 7A). Moreover, we observed clinical correlations between high tdEV_protein_ score and patient survival, including RFS and overall survival (OS) (Figure 7B). To confirm the impact of altered ECM1, MBL2, BTD and RAB5C expression in existing clinical patient studies, we analysed four biomarker‐altered and non‐altered groups with a survival of 1084 patients in the pan‐cancer atlas (Figure 7C) and 2509 patients in the METABRIC data (Figure 7D). We found that the RFS was significantly lower in the group with altered ECM1, MBL2, BTD, and RAB5C expression levels, which is consistent with our results, while OS showed no significance. This might be due to the larger sample size and the possibility that the patients may have received other treatments after recurrence, which could have affected the results.

## 4 Discussion

In this study, we isolated tdEVs using a microfluidic chip‐based protocol and performed an in‐depth comparison of the tdEVs of 100 patients with BC and 30 healthy women to obtain critical, high‐purity tumoUr proteomic information (Figure S14). We found that the immuno‐affinity isolation method reduced the contaminants compared to the PEG isolation method. Isolating tdEVs using a microfluidic chip‐based protocol facilitated the removal of contaminants such as albumin and immunoglobulins and provided important high‐purity tumour proteomic information. We used the LSBoost‐CNN‐SVM algorithm to predict the optimal biomarkers using ML methods (Mao et al. 2005; Han et al. 2012; Lee et al. 2023, Belayneh et al. 2016; Park et al. 2023). To improve the learning reliability, we applied the hybrid LSBoost algorithm, combining the advantages of both AdaBoost and gradient boost models to build the weight equation before classification. Subsequently, the CNN‐SVM algorithm was applied, which integrates the capability of the CNN model for large‐scale data classification with multiple SVM classifiers, thereby classifying proteins based on weight order (Figure 4). The top 4 EV protein biomarkers listed in weight order, including ECM1, MBL2, BTD, and RAB5C, overlapped with 26 significant biomarkers that distinguished patients with BC from healthy individuals, as identified by mass spectrometer‐based proteomic analysis (Figure 2 and Table 1). This observation suggests that biomarkers identified by the LSBoost‐CNN‐SVM algorithm have the potential for both TNBC diagnosis and recurrence prediction.

According to the achievements made by Vinik et al., FAK, MEK1, and fibronectin were indicated as key indicators for the early detection and therapeutic monitoring of BC (Vinik et al. 2020). In addition, Tian et al. reported that EV PSMA is a significant biomarker for monitoring and prognosis of metastatic BC (Tian et al. 2021). These EV biomarkers were also identified in our study's initial EV proteins but were excluded during the marker optimization process using the LSBoost‐CNN‐SVM algorithm. It implied that the marker selection results may vary depending on the analytical intent of ML. Considering TNBC's aggressive characteristics—such as its limited treatment options, and higher likelihood of recurrence—even though it comprises only about 15% of all breast cancer cases, our study emphasizes the critical need for precise diagnosis and monitoring of TNBC. Indeed, in the United States and Europe, the 5‐year overall survival rate for patients with TNBC is 75%–77%, markedly lower than the 84.8%–93% observed for non‐TNBC cases (Hsu et al. 2022). For those with metastatic TNBC at a high risk of recurrence, the 5‐year survival rate falls dramatically to between 4% and 20%. Therefore, we have focused on identifying unique EV biomarkers to more robustly diagnose TNBC and predict its recurrence.

We analysed the selected four‐protein signature (ECM1, MBL2, BTD and RAB5C) in a proteomic analysis set composed of 43 patients with TNBC and 30 healthy women (Figure S11), followed by an evaluation with an independent validation set composed of 30 patients with TNBC and 10 healthy women (Figure 7A). We confirmed that four EV protein markers are potential biomarkers for BC diagnosis and recurrence prediction. Some reports have suggested that elevated levels of ECM1 and MBL2 play important roles in cancer development and progression, implying their prospective utility as biomarkers for identifying high‐risk patients and as prognostic tools for clinicians. Notably, ECM1 is known to activate endothelial cells, boosting angiogenesis, which, in turn, fosters tumour growth and metastasis (Lee et al. 2015; Wang et al. 2003, 2019; Xiong et al. 2012). In addition to its general influence on metastasis in other cancers, the overexpression of ECM1 specifically augments the invasive capacity and metastatic potential of BC cells (Han et al. 2001; Gomez‐Contreras et al. 2017). Ongoing research on ECM1 within EVs is promising (Niu et al. 2019); nevertheless, more studies are required to establish its role as a therapeutic target or prognostic marker. Similarly, Aykut et al. reported that MBL2 induces cancer progression in pancreatic cancer patients (Aykut et al. 2019). Moreover, MBL2, an integral component of the innate immune system, potentially modulates immunological counteractions against tumour cells (Bernig et al. 2007).

Although RAB5C and BTD have been mentioned in certain cancer studies (Baptistella et al. 2019; Onodera et al. 2012; Wang et al. 2023), they may not yet be standard or widely accepted markers for BC detection or recurrence. RAB5C, a member of the RAS oncogene family, is recognized for its involvement in fundamental cellular activities such as endocytosis and vesicle transport, and is speculated to play a role in EV release (Blanc and Vidal 2018). We found contrasting results regarding BTD compared to previous studies showing decreased BTD levels in tissues and blood, however, we believe this is because we specifically studied tdEVs in the blood. For example, So et al. found that BTD, a protein involved in the recycling of biotin, was decreased in the nuclei of thyroid cancer patients but showed an increase in the cytosol (So et al. 2012). This finding contradicts previous beliefs that BTD levels are diminished in the bloodstream and proposes that BTD from the cytoplasm may be released into the bloodstream through EVs. The notable increase of BTD in EVs from breast cancer patients highlighted in our study is an important advancement in comparing BTD levels in the bloodstream to those in EVs derived from blood. This comparison can provide further understanding of the connection between biotin recycling and cancer. Furthermore, BTD is postulated to influence EV formation through its effect on acetyl‐CoA dynamics, which subsequently affect cholesterol synthesis (Skotland et al. 2017; Wang et al. 2020). Considering their biological functions (Hymes and Wolf 1999; Meléndez 2000; Bucci et al. 1992), RAB5C and BTD may simultaneously play pivotal roles in facilitating EV‐mediated exchanges, thereby potentially influencing the interactions between cancer cells and their microenvironment, which could affect tumour progression and recurrence. Despite this rationale, the precise roles of these markers in BC require further clinical validation.

Research into the clinical significance of EV proteins has been rapidly expanding, with increasing emphasis on their fundamental roles in disease processes (Bandu et al. 2024). While biomarkers such as ctDNA, mRNA, miRNA, and CTC are also invaluable for breast cancer diagnosis, tdEVs offer distinct advantages. For instance, ctDNA provides insights into tumour‐specific genetic alterations, mRNA reflects gene expression patterns, and CTCs offer information about metastasis, all of which facilitate cancer diagnostics (Stergiopoulou et al. 2023; Connal et al. 2023). However, tdEVs offer a broader perspective by not only carrying protein cargo but also actively transferring their contents to neighbouring cells through paracrine signalling, to themselves via autocrine signalling, or to distant regions through endocrine signalling within the tumour microenvironment (van Niel et al. 2022). This capability allows tdEVs to mediate complex interactions between tumours and their surrounding cells, offering a more comprehensive understanding of tumour behaviour. Additionally, tdEVs can be sorted based on specific antigens, enhancing their versatility and potential as diagnostic tools. This approach allows for the detection of tumour‐specific markers with clinical relevance, which, when combined with other biomarkers such as ctDNA and mRNA, can provide deeper insights into cancer progression and therapeutic outcomes. Consequently, tdEV‐based diagnostics have the potential to complement traditional methods, improving diagnostic accuracy and efficiency, particularly within personalized, multi‐marker strategies.

Notably, protein markers identified in tdEVs were also detected in tissue‐based assays, further supporting their potential as diagnostic biomarkers. IHC analysis of paired tumour tissue samples from 10 patients confirmed the expression of four markers (BTD, ECM1, MBL2, and RAB5C) in both blood and tissues, as demonstrated in our study (Figure S15A). Under pathological evaluation, BTD exhibited consistent expression in 100% (10/10) of patients, ECM1 in 70% (7/10), MBL2 in 40% (4/10), and RAB5C in 100% (10/10), with signal intensity varying from weak to strong (Figure S15B). Importantly, recent studies have highlighted that EVs mirror the molecular characteristics of their tissue of origin, reinforcing the relevance of these markers in capturing tumour‐specific information. Collectively, these findings underscore the clinical potential of tdEVs in providing valuable insights into tumour biology and advancing non‐invasive diagnostic strategies.

In our analysis of multiple EV protein markers, we observed that an increased number of biomarkers led to an increased AUC and statistical significance (Figure S16). Individual protein marker analysis showed that BTD exhibited the highest AUC, followed by RAB5C, ECM1, and MBL2 in diagnosing TNBC. Notably, our data confirms that incorporating all four biomarkers yields superior performance in both detecting cancer and forecasting prognosis. Given the limited resources, it is imperative to construct a biomarker panel with the least number of necessary markers to ensure that they function synergistically. More protein markers may indeed enhance diagnostic accuracy and outcome predictions; however, determining the optimal combination of biomarkers for our cancer diagnostic assay required a careful balance between improving diagnostic performance and managing its complexity and costs. Adding more biomarkers could theoretically improve the sensitivity and specificity of the assay. However, there is also the risk of increased complexity and variability in results. Future developments and technologies may allow us to include more markers without compromising these concerns, at which point we will consider expanding the number of biomarkers.

Our ML‐reinforced microfluidic platform demonstrated robust performance in diagnosing BC and predicting its recurrence using 170 plasma samples, representing a significant advancement in BC diagnostics. However, our study has several limitations. First, the restricted sample collection from a single research group limits the generalizability of our findings, emphasizing the need for studies involving diverse cohorts to capture broader variability. Second, the variation observed in the detection of ER‐associated proteins, including calnexin (CANX) and mitochondrial cytochrome c (CYCS), and in the expression of EV markers, as demonstrated by LC‐MS/MS analysis (Figure S5), may be attributed to the inherent diversity of EVs, as noted in MISEV2023 (Welsh et al. 2024). However, the possibility of contamination by non‐EV components cannot be completely ruled out. Nevertheless, the breast cancer‐associated EV markers—EpCAM and CD49f— demonstrate strong potential in distinguishing tdEVs from contaminants within the heterogeneous EV pool. While not entirely ‘breast cancer‐specific,’ these markers likely reflect tumour characteristics effectively. Identifying additional markers with greater tumour specificity through comparisons with other cancer types will be crucial to further refine our platform. Moreover, public data reveal that these markers are also expressed in other cancers (Figure S17), underscoring the need for further research to enhance specificity. Lastly, exploring efficient biomarker combinations remains critical. As patient data expand, iterative improvements to our ML algorithm will enhance diagnostic accuracy, ultimately boosting the platform's overall predictive power.

In conclusion, while our multi‐marker sensing platform shows promising advancements in BC screening and prognosis, particularly for TNBC, it is important to approach these findings with caution. Although the platform demonstrates the potential for offering a faster, more accurate, and potentially cost‐effective solution compared to traditional diagnostic methods, the observed correlations between the identified biomarkers and cancer aggressiveness do not establish a direct cause‐and‐effect relationship. Rigorous validation with larger and more diverse datasets is necessary, and further research is required before these biomarkers can be routinely implemented in clinical settings.

## Author Contributions

**Ju‐yong Hyon**: Investigation (equal); writing ‐ original draft (equal). **Min Woo Kim**: Investigation (equal); writing ‐ original draft (equal). **Kyung‐A Hyun**: Investigation (equal); writing ‐ original draft (equal). **Yeji Yang**: Investigation (supporting). **Seongmin Ha**: Investigation (supporting); methodology (equal). **Jee Ye Kim**: Investigation (supporting); methodology (equal). **Young Kim**: Investigation (supporting). **Sunyoung Park**: Investigation (supporting). **Hogyeong Gawk**: Investigation (supporting). **Heaji Lee**: Investigation (supporting). **Suji Lee**: Investigation (supporting). **Sol Moon**: Investigation (supporting). **Eun Hee Han**: Investigation (supporting). **Jin Young Kim**: Investigation (supporting). **Ji Yeong Yang**: Validation (supporting). **Hyo‐Il Jung**: Conceptualization (equal); funding acquisition (equal); supervision (equal); writing ‐ review and editing (equal). **Seung Il Kim**: Conceptualization (equal); funding acquisition (equal); writing ‐ review and editing (equal). **Young‐Ho Chung**: Conceptualization (equal); funding acquisition (equal); supervision (equal); writing ‐ review and editing (equal).

## Conflicts of Interest

The authors declare no conflicts of interest.

## Supporting information



Supporting Information

Supporting Information

Supporting Information

## Data Availability

All data required to evaluate the conclusions in the paper are presented in the paper and/or Supplementary Materials. Additional data related to this study can be obtained from the authors. Requests for the data should be submitted to chungyh@kbsi.re.kr. The raw MS proteomic data and complete MaxQuant search results were deposited in the ProteomeXchange Consortium (www.proteomexchange.org/) via the PRIDE partner repository with the dataset identifier PXD047470. Spectra identifying modified peptides and proteins based on single‐peptide matches can be viewed in the MaxQuant Viewer included in the MaxQuant software, which was also deposited in the same location.
