# Supplementary material for: Extracellular Vesicle Proteome Analysis Improves Diagnosis of Recurrence in Triple‐Negative Breast Cancer
Source: J Extracell Vesicles. 2025 Jun 23;14(6):e70089. doi: 10.1002/jev2.70089 (PMC12183389; doi:10.1002/jev2.70089)
Supplement: Supplementary file 1 — Supporting Information [file JEV2-14-e70089-s002.docx]

Supplementary Information

**Extracellular vesicle proteomic analysis improves diagnosis of recurrence in triple-negative breast cancer**

Ju-yong Hyon,^1†^ Min Woo Kim,^2†^ Kyung-A Hyun,^3†^ Yeji Yang,^1,4,5^ Seongmin Ha,^6^ Jee Ye Kim,^2^ Young Kim,^2^ Sunyoung Park,^6,7^ Hogyeong Gawk,^6^ Heaji Lee,^1^ Suji Lee,^2^ Sol Moon,^2^ Eun Hee Han,^8,9^ Jin Young Kim,^1,4^, Ji Yeong Yang^6,7^, Hyo-Il Jung,^6,7*^ Seung Il Kim,^2*^ Young-Ho Chung^1,10*^

^1^ Research Center for Digital Omics, Korea Basic Science Institute, 162, Yeongudanji-ro, Ochang-eup, Cheongwon-gu, Cheongju-si, Chungcheongbuk-do, 28119, Republic of Korea. [hjy1234@kbsi.re.kr](mailto:hjy1234@kbsi.re.kr) (J-YH); [yangyj@kbsi.re.kr](mailto:yangyj@kbsi.re.kr) (YY); [heaji1207@kbsi.re.kr](mailto:heaji1207@kbsi.re.kr) (HL); [jinyoung@kbsi.re.kr](mailto:jinyoung@kbsi.re.kr) (JiYK); [chungyh@kbsi.re.kr](mailto:chungyh@kbsi.re.kr) (JHC)

^2^ Department of Surgery, Yonsei University College of Medicine, 50-1 Yonsei-ro, Seodaemun-gu, Seoul, 03722, Republic of Korea. [minwookim@yuhs.ac](mailto:minwookim@yuhs.ac) (MWK); [JEEYE0531@yuhs.ac](mailto:JEEYE0531@yuhs.ac) (JeYK); [martino.young1@gmail.com](mailto:martino.young1@gmail.com) (YK); [SOOJEE2@yuhs.ac](mailto:SOOJEE2@yuhs.ac) (SL); [sol9912@yuhs.ac](mailto:sol9912@yuhs.ac) (SM); [SKIM@yuhs.ac](mailto:SKIM@yuhs.ac) (SIK)

^3^ School of Biopharmaceutical and Medical Sciences, Sungshin Women’s University, 55 Dobong-ro, 76 ga-gil, Gangbuk-gu, Seoul, 01133, Republic of Korea. [kahyun@sungshin.ac.kr](mailto:kahyun@sungshin.ac.kr) (K-AH)

^4^ Critical Diseases Diagnostics Convergence Research Center, Korea Research Institute of Bioscience and Biotechnology, 125 Gwahak-ro, Yuseong-gu, Daejeon, 34141, Republic of Korea.

^5^ Department of Biological Sciences, Korea Advanced Institute for Science and Technology (KAIST), Daejeon, 34141, Korea

^6^ School of Mechanical Engineering, Yonsei University, 50 Yonsei-ro, Seodaemun-gu, Seoul, 03722, Republic of Korea. [choosesay@naver.com](mailto:choosesay@naver.com) (SH); [angelsy88@gmail.com](mailto:angelsy88@gmail.com) (SP); [kirapi83@gmail.com](mailto:kirapi83@gmail.com) (HG); [yjy9006@naver.com](mailto:yjy9006@naver.com) (JY); [uridle7@yonsei.ac.kr](mailto:uridle7@yonsei.ac.kr) (HIJ)

^7^ The DABOM Inc., 50 Yonsei-ro, Seodaemun-gu, Seoul, 03722, Republic of Korea.

^8^ Biopharmaceutical Research Center, Korea Basic Science Institute, 162, Yeongudanji-ro, Ochang-eup, Cheongwon-gu, Cheongju-si, Chungcheongbuk-do, 28119, Republic of Korea. [heh4285@kbsi.re.kr](mailto:heh4285@kbsi.re.kr) (EHH);

^9^ Department of Bio-Analytical Science, University of Science and Technology, 217, Gajeong-ro, Yuseong-gu, Daejeon 34113, Republic of Korea.

^10^ Department of Analytical Science and Technology, Graduate School of Analytical Science and Technology (GRAST), Chungnam National University, 99, Daehak-ro, Yuseong-gu, Daejeon 34134, Republic of Korea.

^†^ All authors contributed equally to this work.

* Corresponding authors:

chungyh@kbsi.re.kr (Y. H. Chung); SKIM@yuhs.ac (S. I. Kim), uridle7@yonsei.ac.kr (H. I. Jung)

**This file includes:**

Figs. S1–S17

Tables S1–S5


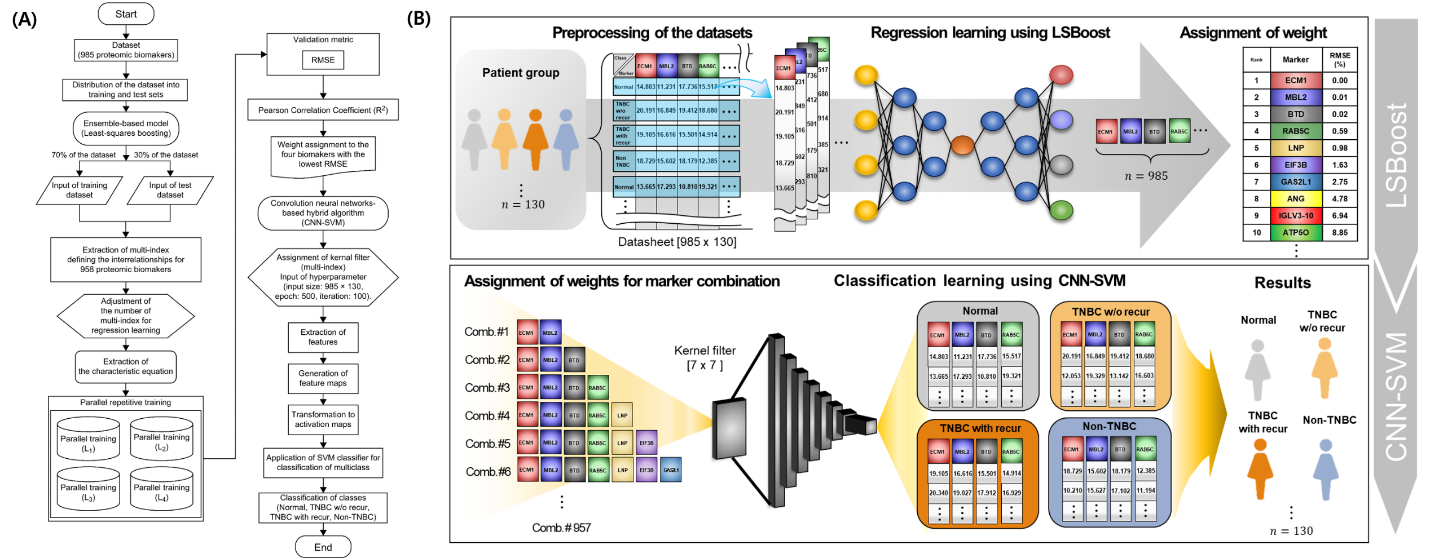


Fig. S1. Flow chart of the hybrid machine learning algorithm (LsBoost-CNN-SVM). RMSE, root mean square error; TNBC, triple negative breast cancer; CNN, convolutional neural network; SVM, support vector machine.


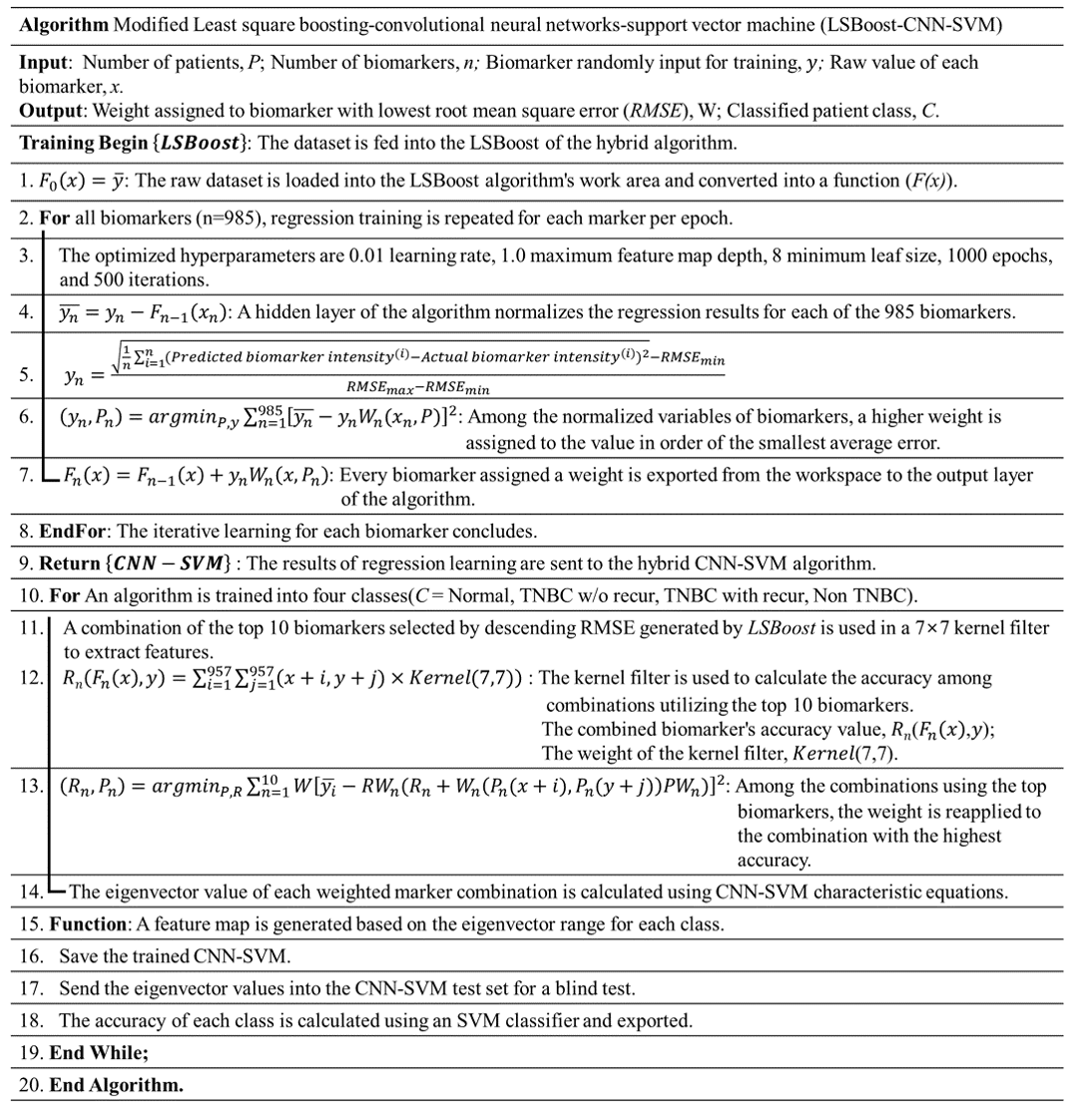


**Fig. S2. Working process with summarized code of hybrid algorithm**


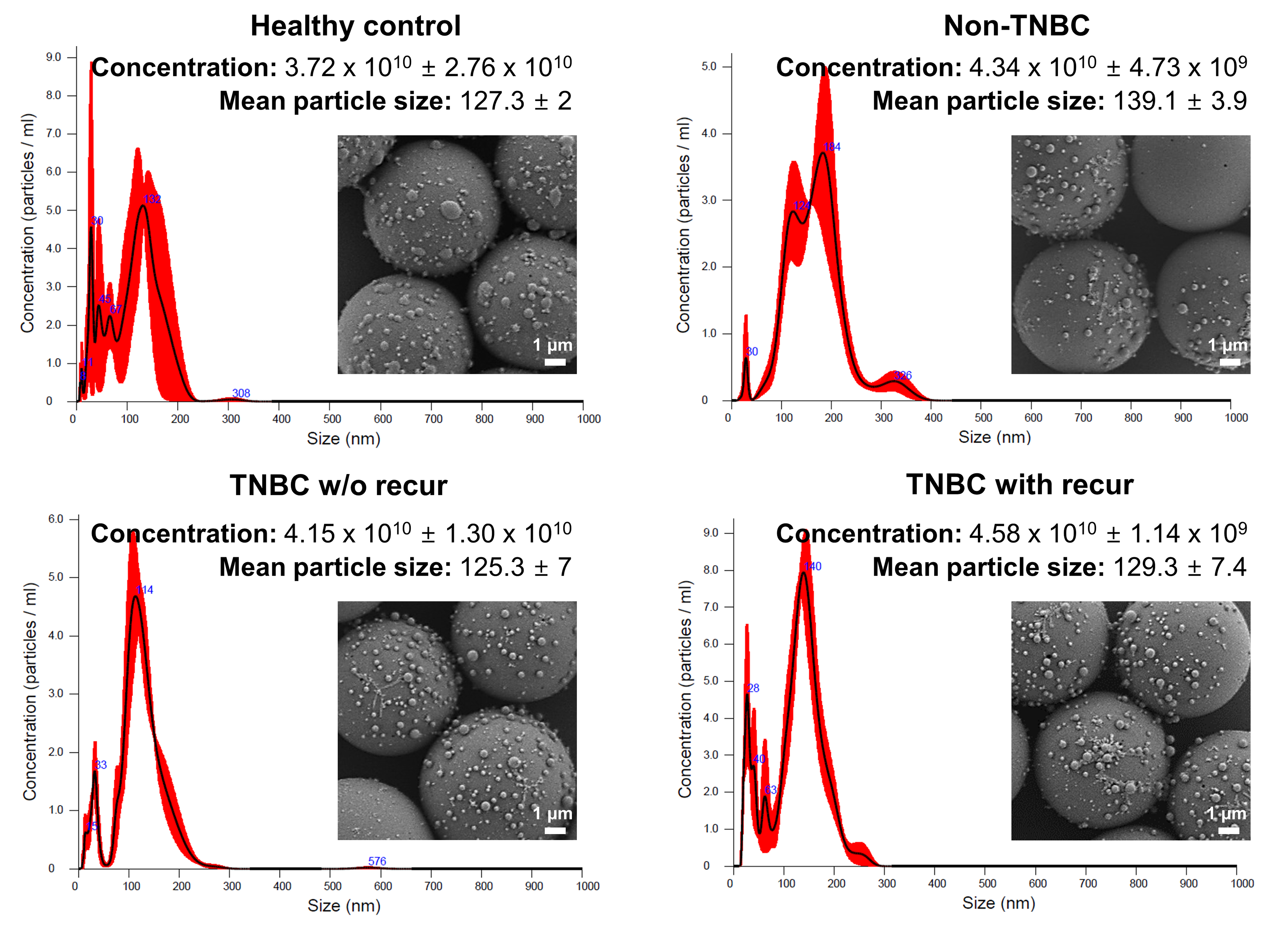


**Fig. S3.** **Characterization of tdEVs from plasma samples of different groups using NTA and SEM.** NTA size distribution profiles and SEM imaging of tdEVs isolated from plasma samples of representative individuals from four groups: Healthy control, Non-TNBC, TNBC w/o recur, and TNBC with recur. NTA measurements were performed in triplicate, and size distribution values are expressed in nanometers (nm). Concentration values are presented as particles per mL (particle/mL). SEM images confirm the presence of spherical vesicles attached to microbeads, consistent with the typical morphology of EVs. Scale bar: 1 µm.


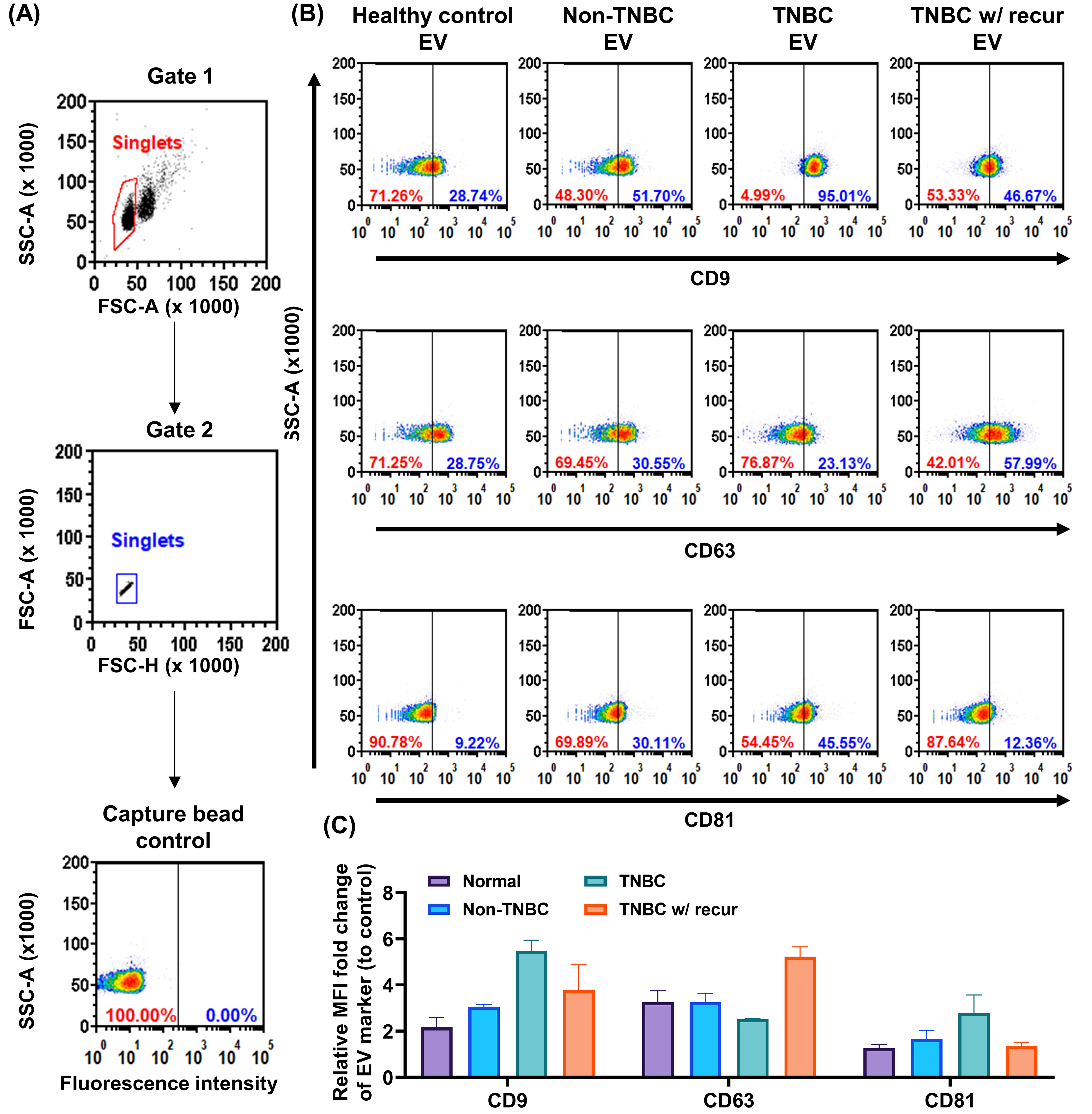


**Fig. S4. Flow cytometry analysis of tdEV surface marker expression.** (A) Gating strategy for identifying singlet beads to exclude doublets and aggregates. The control gating with capture beads demonstrates the absence of fluorescence signals in the negative control. (B) Comparison of fluorescence intensity shifts for EV surface markers (CD9, CD63, and CD81) across four groups: Healthy control EV, Non-TNBC EV, TNBC w/o recur EV, and TNBC with recurrence EV. (C) Quantification of mean fluorescence intensity (MFI) fold change for each marker (CD9, CD63, CD81) relative to the capture bead control.


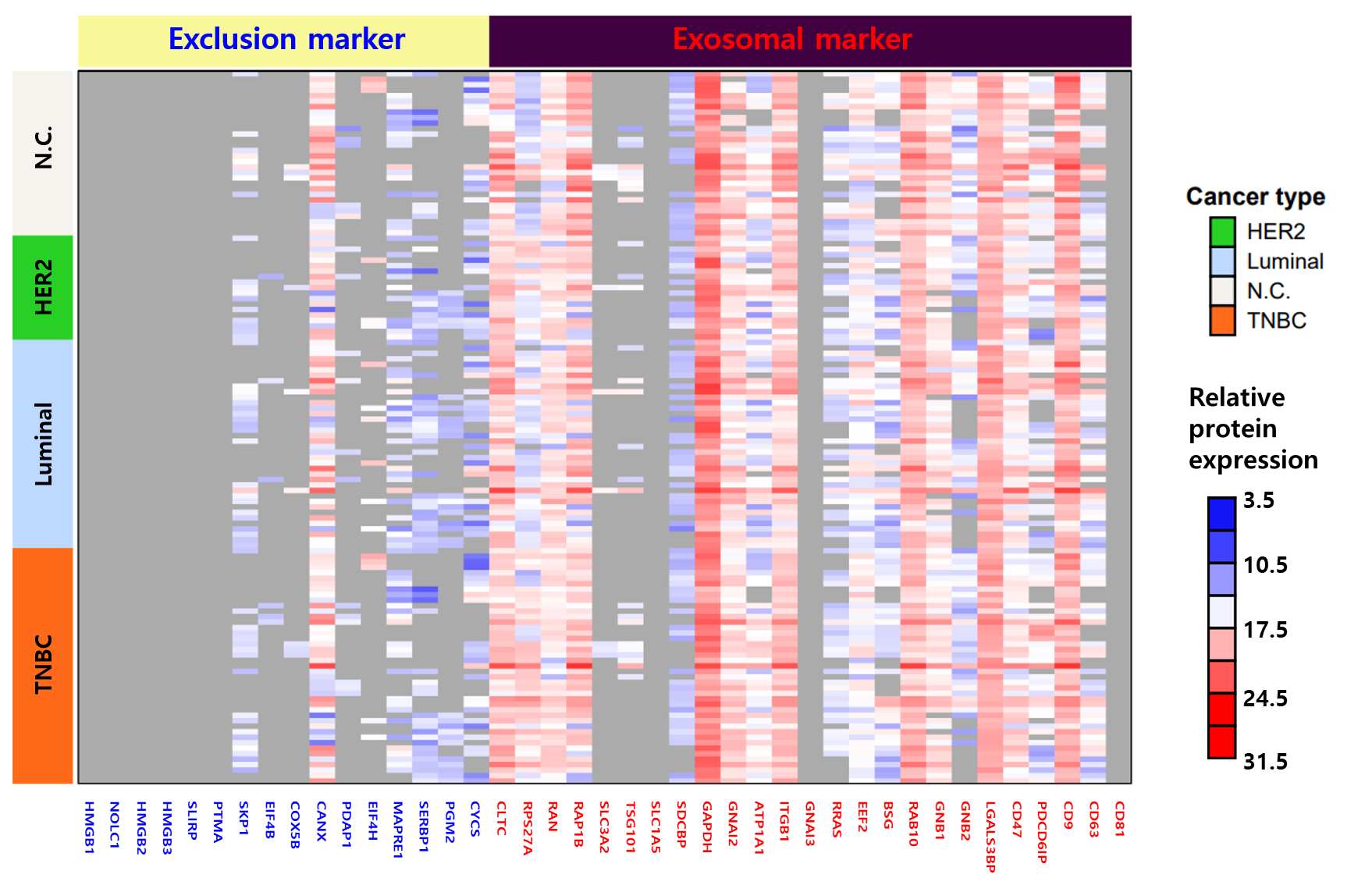


**Fig. S5.** **Quantitative proteomics analysis of tdEVs identifies exosomal biomarkers and exclusion proteins.** Heatmap of exosomal biomarker proteins (red text) and exclusion biomarker proteins (blue text). In this panel, individual entities are labeled with two color codes. The first color represents the breast cancer subtype (HER2 is green, luminal is light blue, N.C. is grey, and TNBC is orange). The second color codes the relative protein expression.


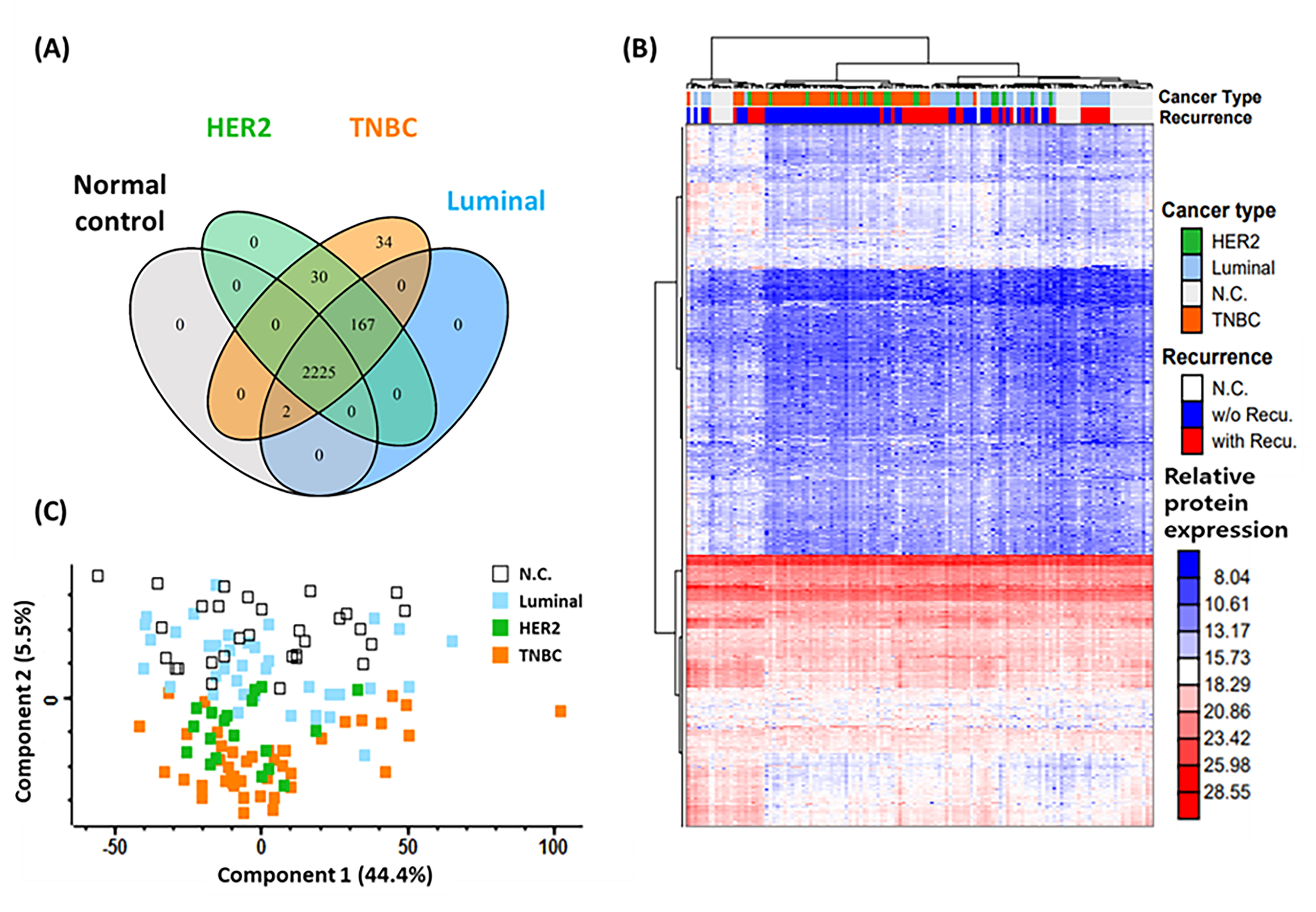


**Fig. S6. Overview of quantitative proteomics analysis of tumor-derived EVs.** (A) Venn diagram of tumor-derived EVs in subtypes of breast cancer patients against identified proteins. (B) Heatmap of Euclidean distances and associated hierarchical clustering dendrograms between 985 identified proteins and patients. Individual entities are labeled with two color codes. The first color represents the breast cancer subtype (HER2 is green, luminal is light blue, N.C. is grey, and TNBC is orange). The second color codes the recurrence of cancer patients (white for N.C., blue for BC w/o recur, and red for BC with recurrence). (C) PCA plot of tdEVs in subtypes of breast cancer patients against 985 identified proteins. N.C., normal control; BC, breast cancer; TNBC, triple negative breast cancer; EV, extracellular vesicle; tdEV, tumor-derived extracellular vesicle; PCA, principal component analysis.


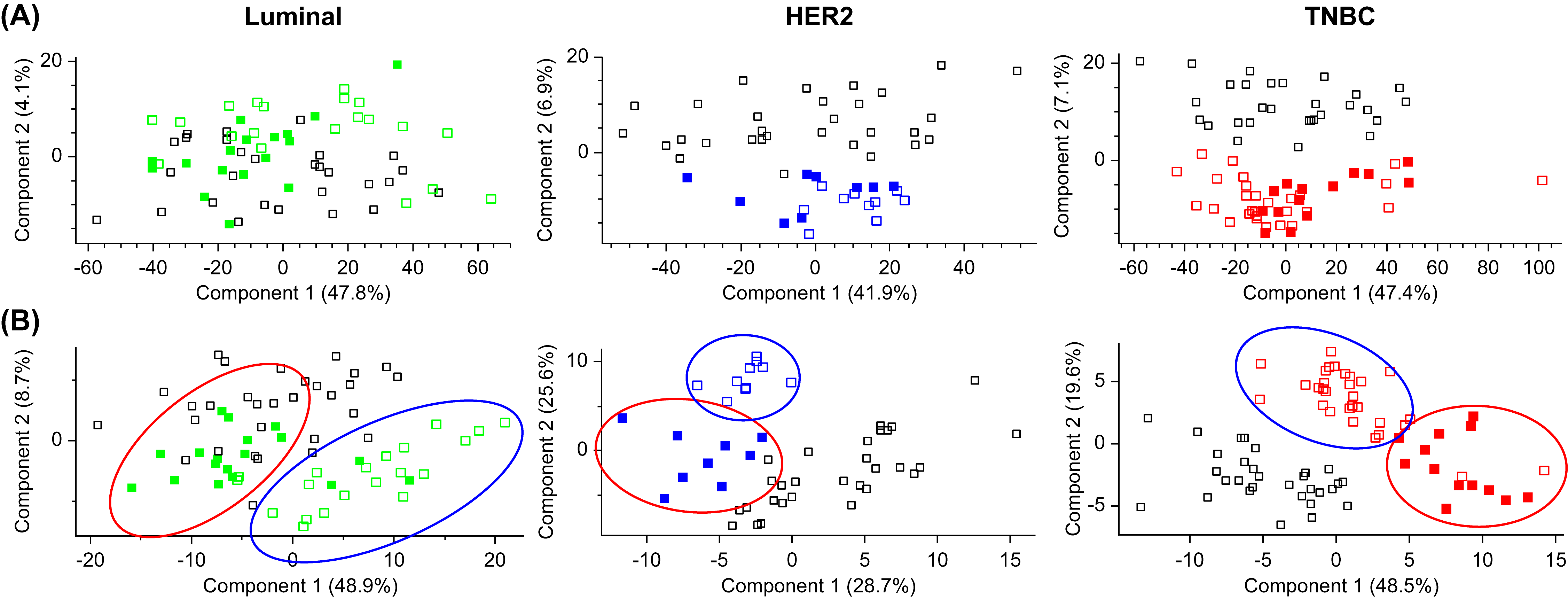


**Fig. S7. PCA plot of the tumor-derived EVs in the subtypes of breast cancer patients against the 985 identified proteins (A) and each of the DEPs (B) in the subtypes of breast cancer.** The red circles cluster patients who relapsed, while the blue circles indicate patients who did not relapse. In each graph, grey squares represent normal people, green for luminal, blue for HER2, and red for TNBC, with filled squares representing recurrent patients and empty squares representing patients without recurrence. PCA, principal component analysis; EV, extracellular vesicle; DEP, differentially expressed proteins; TNBC, triple negative breast cancer.


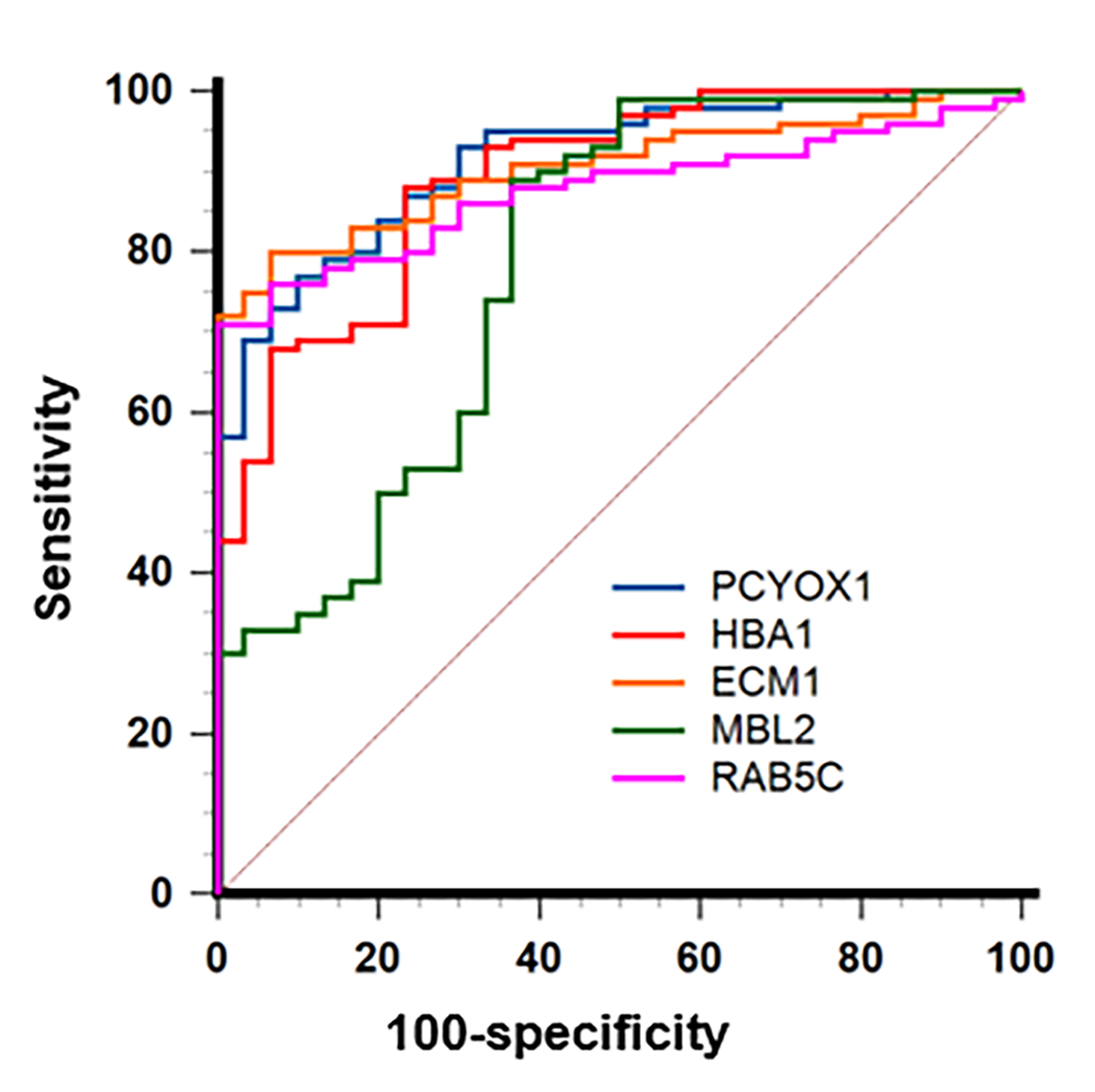


**Fig. S8. Sensitivity and specificity plot of each biomarker.** Five EV protein markers with the highest p-values among the 26 significant proteins to distinguish between healthy controls and patients with breast cancer.


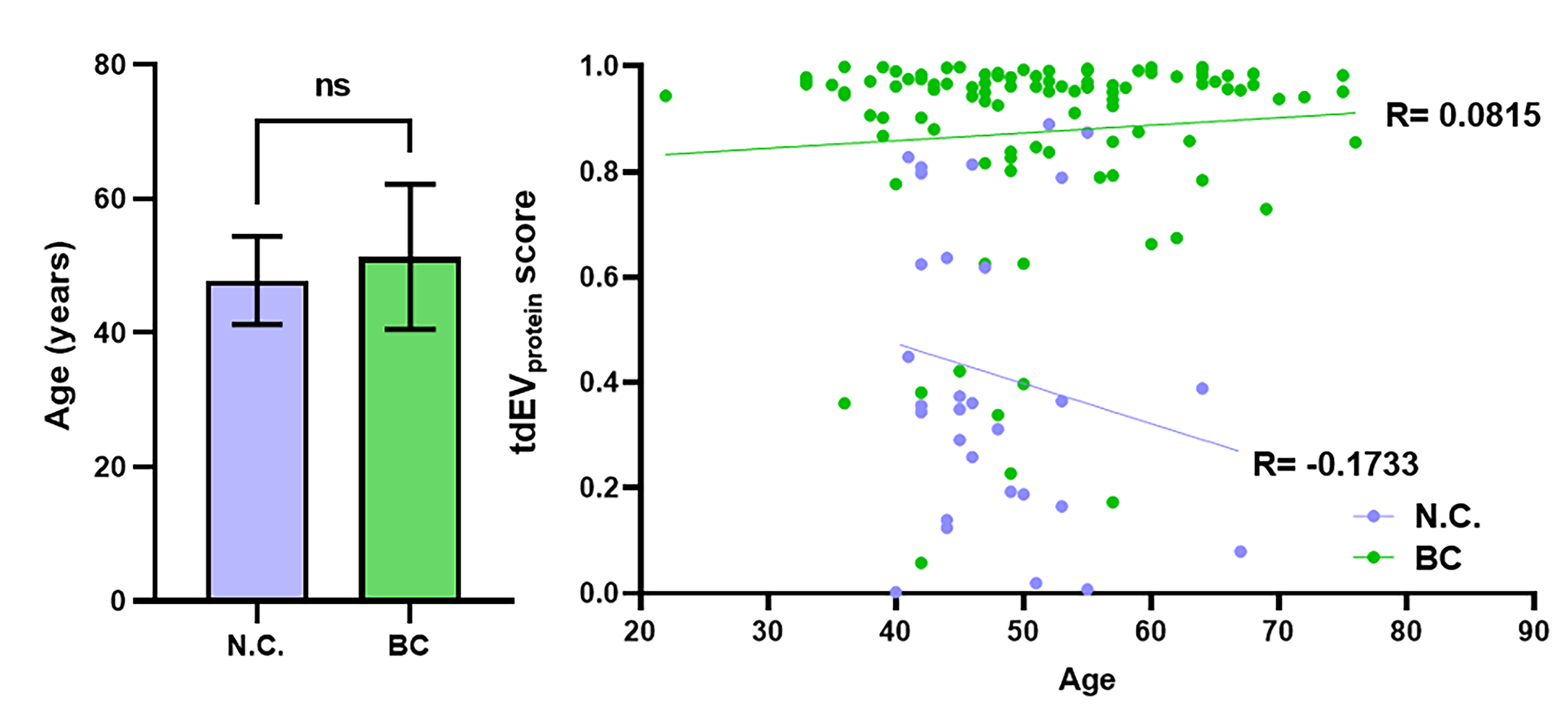


**Fig. S9. Comparison of the effects of age across subjects.** Statistical analyses between N.C. and BC are performed using an unpaired Student’s t-test. Pearson's correlation analysis is performed to analyze the correlation between the tdEV_protein_ score and age distribution. ns, not significant; N.C., normal control; BC, patients with breast cancer; R, Pearson's correlation coefficient.


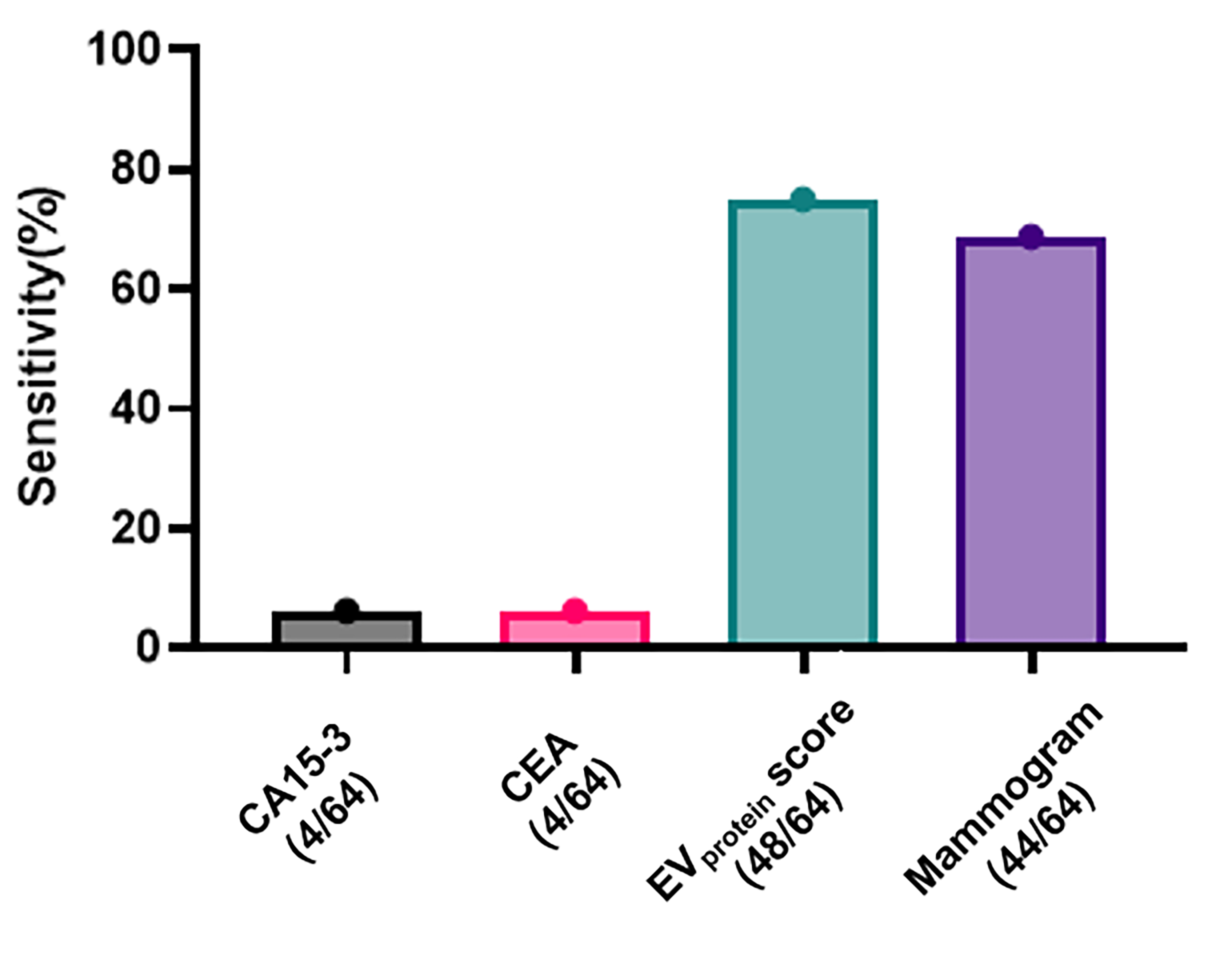


**Fig. S10. Comparative analysis of the sensitivity of conventional diagnostic methods and established EV_protein_ score.** Only 64 patients with BC having all data for the four groups are compared and analyzed. The results show that our approach has higher sensitivity than CA15-3, CEA, and mammography.


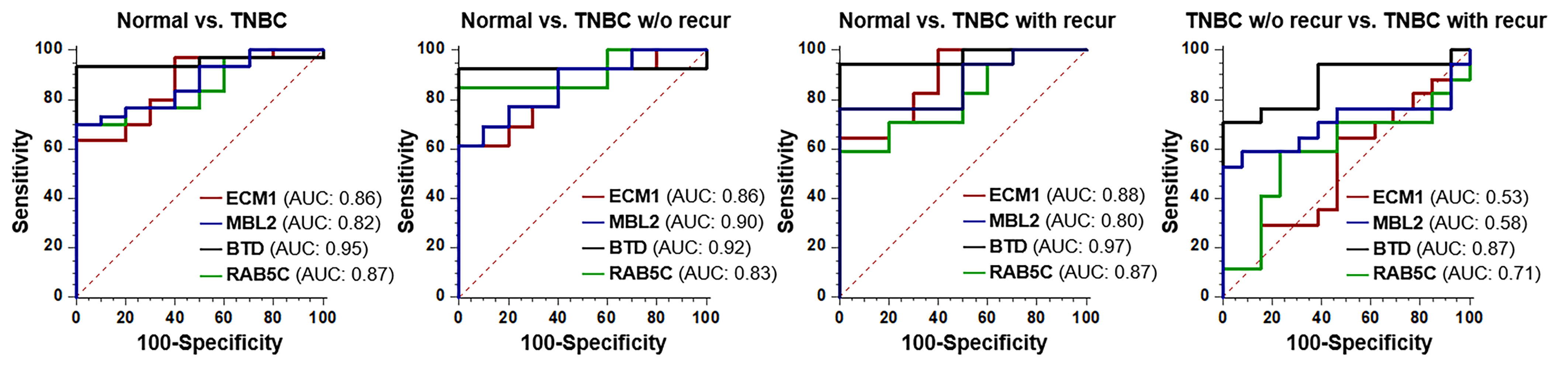


**Fig. 11. ROC analysis and AUC values of each biomarker.** ROC curves of each single tumor-derived EV protein marker to differentiate between N.C. and TNBC, as well as TNBC without recurring and TNBC recurring. The AUC values are calculated using the Wilcoxon/Mann–Whitney test. TNBC, triple negative breast cancer; EV, extracellular vesicle; ROC, receiver operating characteristic; AUC, area under the ROC curve.


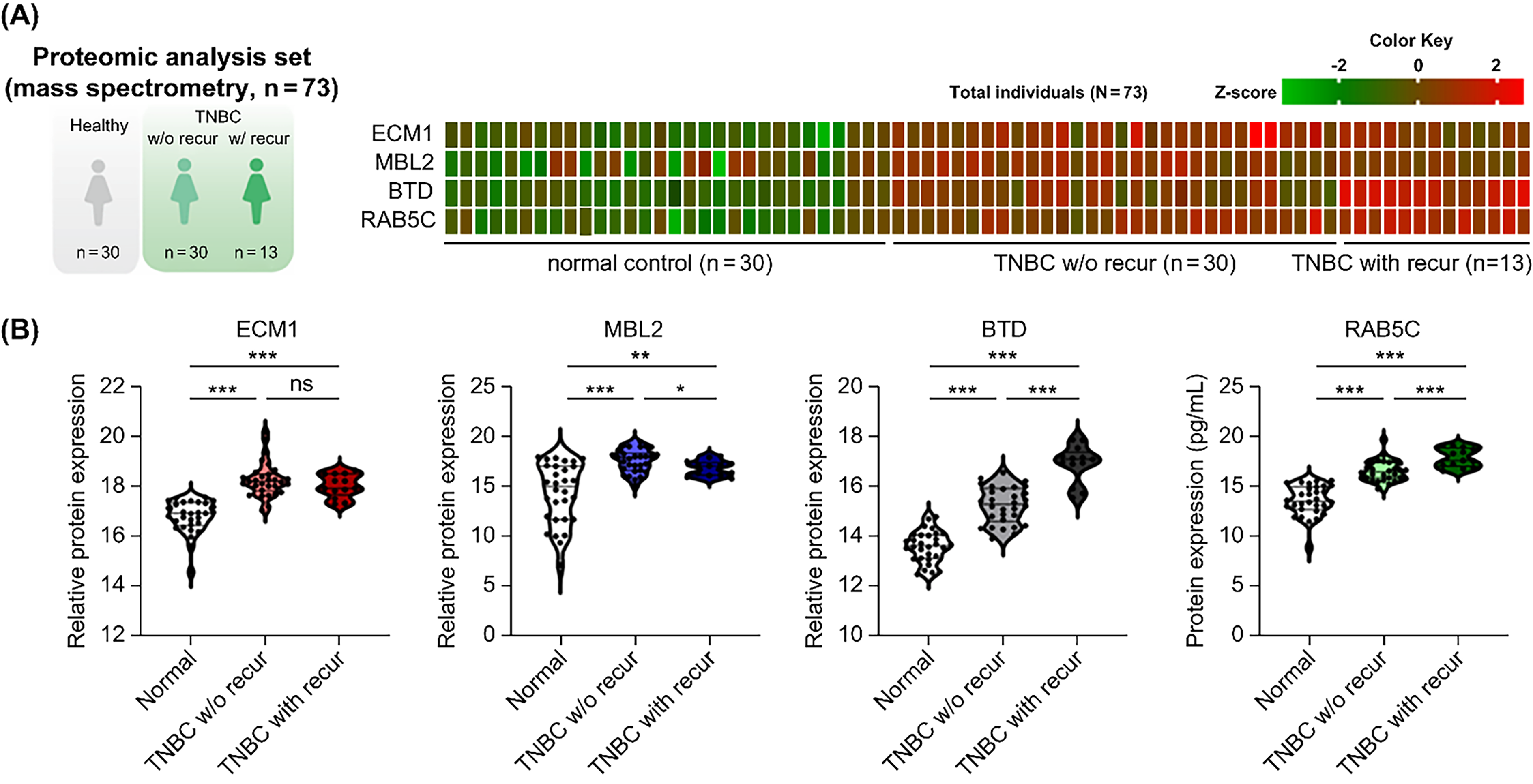


**Fig. S12. Comparative protein expression analysis of the top four predicted tumor-derived EV protein markers after ML analysis in patients with TNBC with and without recurrence.** (A) Heatmap illustrating the protein expression of tumor-derived EV protein markers from patients with TNBC without recurrence (w/o recur) and patients without TNBC with recurrence (with recur) compared with normal controls (NCs). The color key represents the Z-score for protein expression intensity. (B) Comparison of the protein expression levels of tumor-derived EV protein markers in each group including normal controls (n=30), TNBC w/o recur (n=30), and TNBC with recur (n=13). Statistical analyses are performed using one-way ANOVA with Turkey's multiple comparisons between three groups. ns, not significant; *, *p* < 0.05; **, *p* < 0.01; ***, *p* <0.001. TNBC, triple negative breast cancer; EV, extracellular vesicle; ANOVA, analysis of variance.


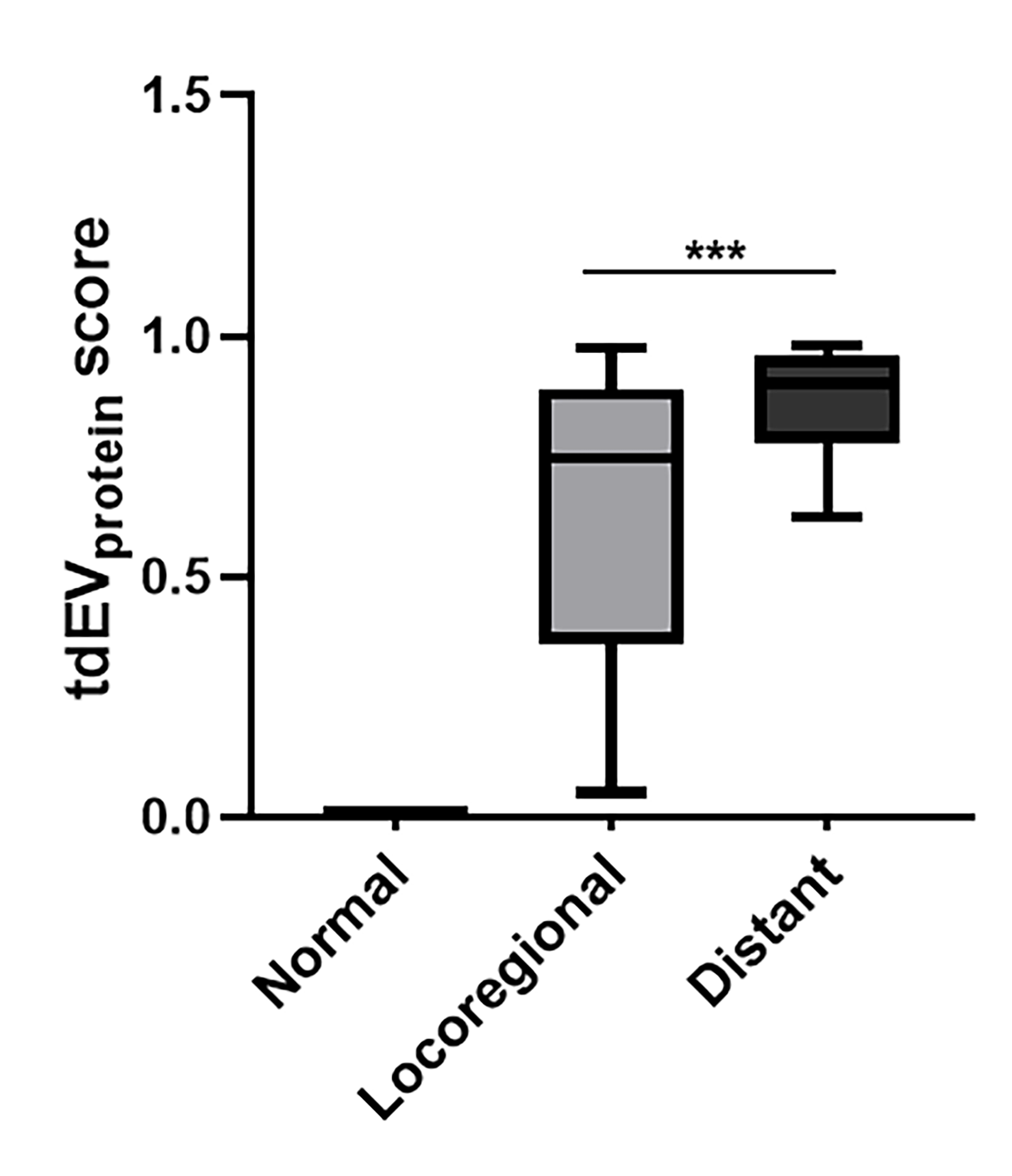


**Fig. S13. Comparison of the effects of recurrence patterns across subjects.** Locoregional recurrence refers to cancer returning to the original site or nearby regions, such as the chest wall or local lymph nodes. Distant recurrence involves cancer spreading to distant parts of the body, like the lungs, brain, bone or liver. Significant differences between groups are determined using one-way ANOVA with Turkey's multiple comparisons. ***, *p* < 0.001. ANOVA, analysis of variance.


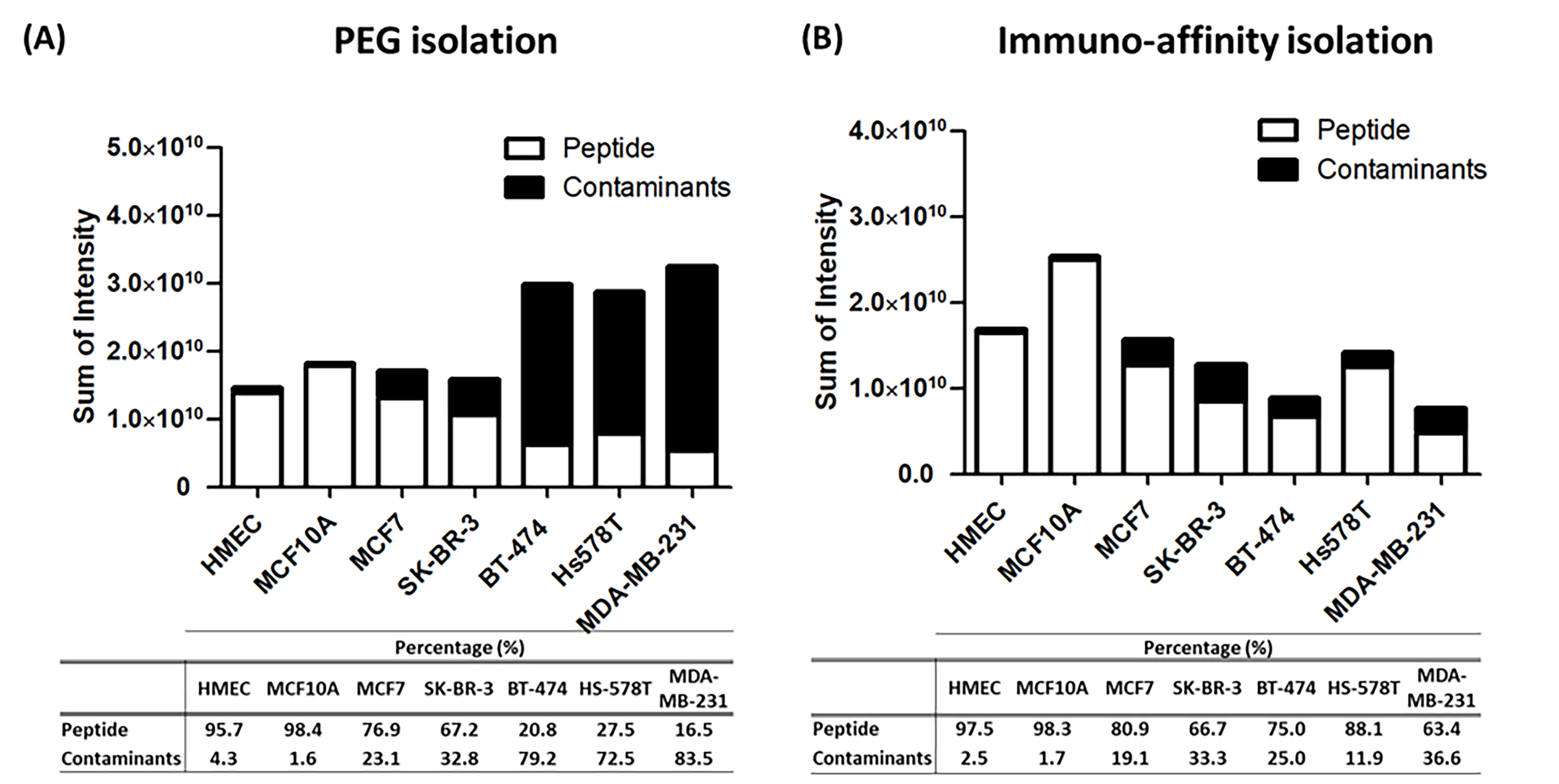


**Fig. S14.** **Comparison of contaminants using different extracellular vesicle isolation methods for different breast cancer cell lines.** (A) PEG isolation method, (B) Immuno-affinity (EpCAM/CD49f) base isolation method. PEG, polyethylene glycol.

**
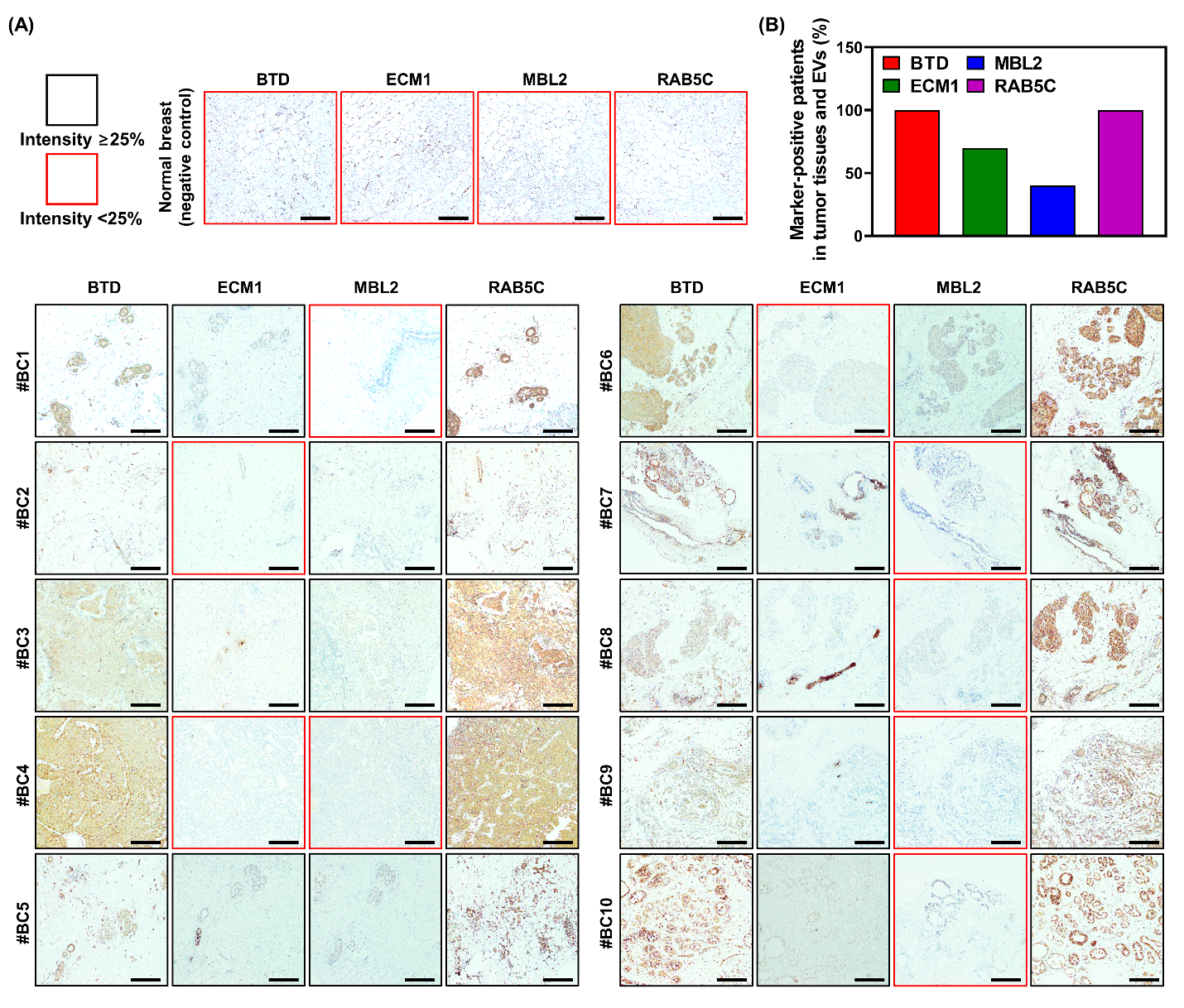
**

**Fig. S15. Protein expression levels of EV protein markers (ECM1, MBL2, BTD, and RAB5C) across clinical breast cancer tissue specimens.** (A) Representative immunohistochemical (IHC) images from patient tissue samples used in our study, showing the expression patterns of each marker (BTD, ECM1, MBL2, and RAB5C) across different specimens. Black squares highlight areas with a strong positive signal (IHC intensity ≥25) for the respective protein, while red squares indicate regions with little or no expression (IHC intensity <25). Adjacent normal breast tissues were used as a negative control for IHC. Original magnification: ×10. Scale bars represent 200 μm. (B) The bar graph shows the proportion of cases where BTD (red), ECM1 (green), MBL2 (blue), and RAB5C (purple) were positively detected in EVs and corresponded with positive IHC staining in tumor tissues (n=10).


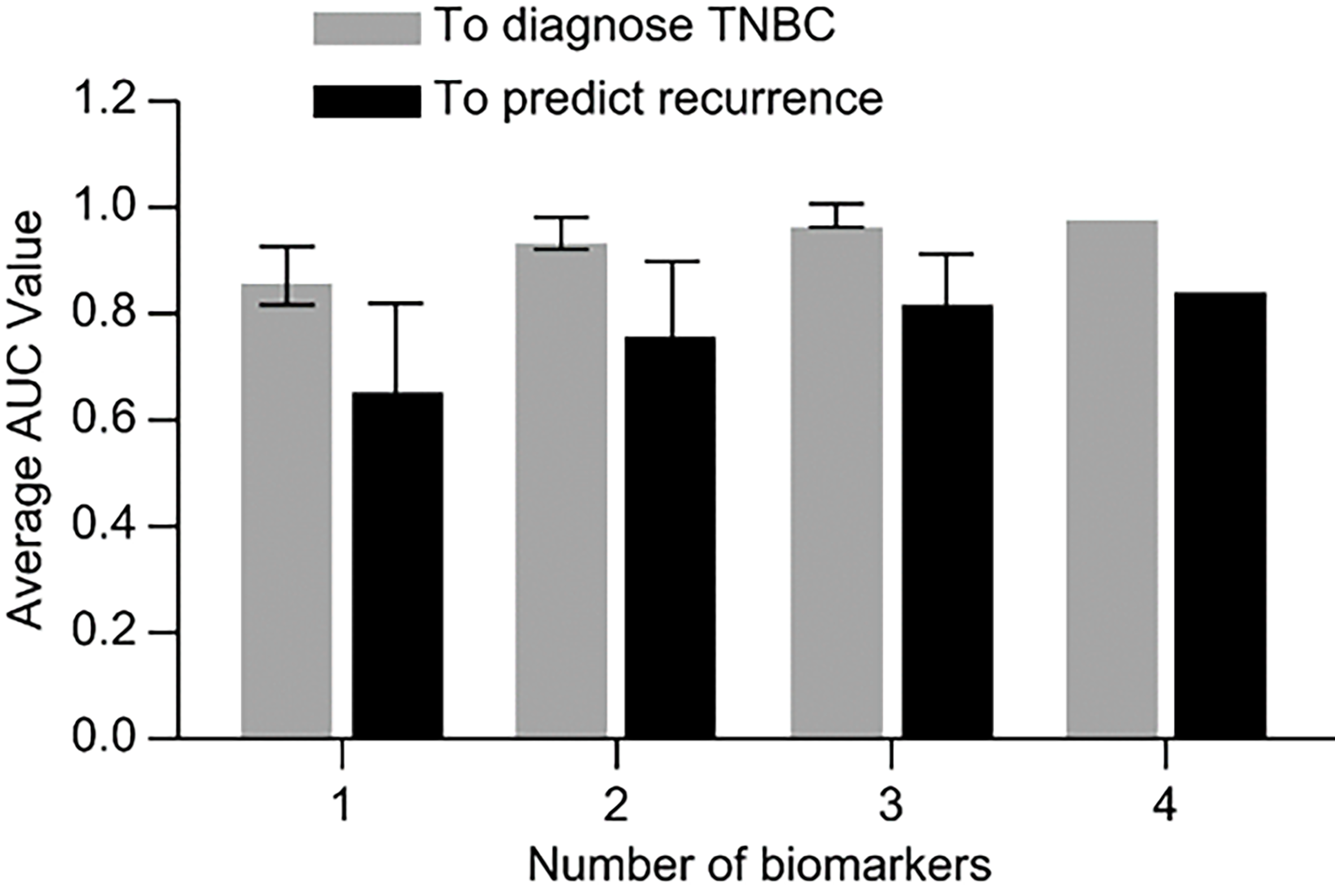


**Fig. S16. Average AUCs with different numbers of EV protein markers.** Error bars represent the standard deviation. AUC, area under the receiver operating characteristic curve; EV, extracellular vesicle; TNBC, triple negative breast cancer.


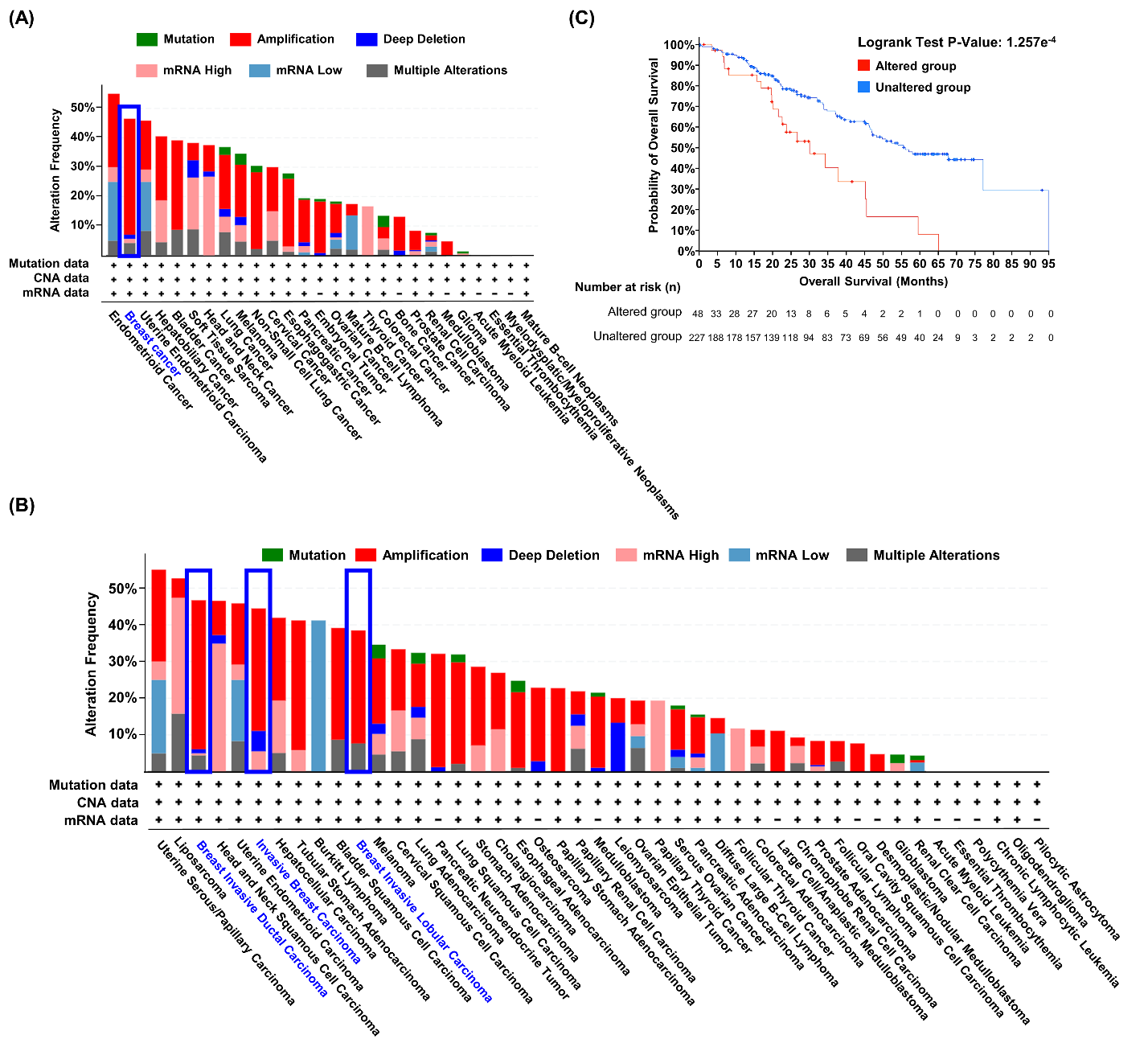


**Fig. S17. Analysis of alterations and survival curves for four biomarkers (ECM1, MBL2, BTD, and RAB5C) in 2,922 pan-cancer patients.** (A) Frequency of alterations in the four biomarkers among 2,922 cancer patients, as studied by the ICGC/TCGA Pan-Cancer Analysis of Whole Genomes Consortium. (B) Frequency of alterations in subgrouped cancer patients. (C) Kaplan-Meier survival curve analysis of pan-cancer patients with alterations in the four biomarkers. The analysis was conducted using cBioPortal software (<http://cbioportal.org>).

Table S1. Clinical characteristics of the enrolled patients

| **Characteristics** | **LC-MS/MS**  **analysis group (n=130)** | **ELISA**  **validation group (n=40)** |
| --- | --- | --- |
| **Subtype** |  |  |
| Luminal | 33 (25.4%) | - |
| HER2 | 24 (18.5%) | - |
| TNBC | 43 (33.1%) | 30 (75%) |
| Normal | 30 (23.0%) | 10 (25%) |
| **Recurrence** |  |  |
| No | 63 (63.0%) | 13 (43.3%) |
| Yes | 37 (37.0%) | 17 (56.7%) |

Table S2. Combination equation for ROC analysis

| **Normal vs. BC (Proteomic set)** | | | | | |
| --- | --- | --- | --- | --- | --- |
| **Variable** | **Biomarker** | | | | **Equation** |
| Combi_1 | ECM1 | MBL2 |  |  | -40.61+2.07*LN(ECM1)+0.37*LN(MBL2) |
| Combi_2 | ECM1 | BTD |  |  | -42.78+2.60*LN(ECM1)-0.08*LN(BTD) |
| Combi_3 | ECM1 | RAB5C |  |  | -36.83+2.01*LN(ECM1)+0.21*LN(RAB5C) |
| Combi_4 | MBL2 | BTD |  |  | -13.08+0.56*LN(MBL2)+0.37*LN(BTD) |
| Combi_5 | MBL2 | RAB5C |  |  | -13.97+0.48*LN(MBL2)+0.51*LN(RAB5C) |
| Combi_6 | BTD | RAB5C |  |  | -9.57+0.10*LN(BTD)+0.63*LN(RAB5C) |
| Combi_7 | ECM1 | MBL2 | BTD |  | -41.55+2.21*LN(ECM1)+0.38*LN(MBL2)-0.12*LN(BTD) |
| Combi_8 | ECM1 | MBL2 | RAB5C |  | -41.55+2.21*LN(ECM1)+0.38*LN(MBL2)-0.12*LN(BTD) |
| Combi_9 | ECM1 | BTD | RAB5C |  | -36.13+1.66*LN(ECM1)+0.37*LN(MBL2)+0.18*LN(RAB5C) |
| Combi_10 | MBL2 | BTD | RAB5C |  | -37.14+2.17*LN(ECM1)-0.23*LN(BTD)+0.28*LN(RAB5C) |
| Combi_11 | ECM1 | MBL2 | BTD | RAB5C | -13.37+0.49*LN(MBL2)-0.10*LN(BTD)+0.032*LN(RAB5C) |
| **Normal vs. TNBC (Immunoassay set)** | | | | | |
| **Variable** | **Biomarker** | | | | **Equation** |
| Combi_1 | ECM1 | MBL2 |  |  | -0.13412+0.0003458*LN(ECM1)-0.0008701*LN(MBL2) |
| Combi_2 | ECM1 | BTD |  |  | -8.76609+0.00013515*LN(ECM1)+0.0051355*LN(BTD) |
| Combi_3 | ECM1 | RAB5C |  |  | -1.87134+0.000011862*LN(ECM1)+0.00059164*LN(RAB5C) |
| Combi_4 | MBL2 | BTD |  |  | -9.38943+0.0037378*LN(MBL2)+0.0053856*LN(BTD) |
| Combi_5 | MBL2 | RAB5C |  |  | -2.07429+0.0014420*LN(MBL2)+0.00060956*LN(RAB5C) |
| Combi_6 | BTD | RAB5C |  |  | -8.88885+0.0045854*LN(BTD)+0.00035673*LN(RAB5C) |
| Combi_7 | ECM1 | MBL2 | BTD |  | -9.39012+0.000010944*LN(ECM1)  +0.0037022*LN(MBL2)+0.0053793*LN(BTD) |
| Combi_8 | ECM1 | MBL2 | RAB5C |  | -2.06259-0.000018952*LN(ECM1)  +0.0014666*LN(MBL2)+0.00061328*LN(RAB5C) |
| Combi_9 | ECM1 | BTD | RAB5C |  | -8.91631+0.000025407*LN(ECM1)  +0.0045869*LN(BTD)+0.00035433*LN(RAB5C) |
| Combi_10 | MBL2 | BTD | RAB5C |  | -9.76186+0.0044356*LN(MBL2)  +0.0048003*LN(BTD)+0.00037130*LN(RAB5C) |
| Combi_11 | ECM1 | MBL2 | BTD | RAB5C | -9.72412-0.00013712*LN(ECM1)+0.0048620*LN(MBL2)  +0.0048337*LN(BTD)+0.00038425*LN(RAB5C) |
| **TNBC with recurrence vs. TNBC w/o recurrence (Immunoassay set)** | | | | | |
| **Variable** | **Biomarker** | | | | **Equation** |
| Combi_1 | ECM1 | MBL2 |  |  | -4.04814+0.0043033*LN(ECM1)+0.032006*LN(MBL2) |
| Combi_2 | ECM1 | BTD |  |  | -510.37408+0.20917*LN(ECM1)+0.26064*LN(BTD) |
| Combi_3 | ECM1 | RAB5C |  |  | -6.84229+0.0044711*LN(ECM1)+0.0016964*LN(RAB5C) |
| Combi_4 | MBL2 | BTD |  |  | -9.52894+0.056082*LN(MBL2)+0.0057696*LN(BTD) |
| Combi_5 | MBL2 | RAB5C |  |  | -5.93773+0.046796*LN(MBL2)+0.0018563*LN(RAB5C) |
| Combi_6 | BTD | RAB5C |  |  | -10.71707+0.0054738*LN(BTD)+0.0017696*LN(RAB5C) |
| Combi_7 | ECM1 | MBL2 | BTD |  | -471.16273+0.17957*LN(ECM1)  +0.45215*LN(MBL2)+0.24099*LN(BTD) |
| Combi_8 | ECM1 | MBL2 | RAB5C |  | -7.31143+0.0029610*LN(ECM1)  +0.033717*LN(MBL2)+0.0017365*LN(RAB5C) |
| Combi_9 | ECM1 | BTD | RAB5C |  | -504.46558+0.16310*LN(ECM1)  +0.23913*LN(BTD)+0.024583*LN(RAB5C) |
| Combi_10 | MBL2 | BTD | RAB5C |  | -487.44898+0.99983*LN(MBL2)  +0.17783*LN(BTD)+0.094106*LN(RAB5C) |
| Combi_11 | ECM1 | MBL2 | BTD | RAB5C | -459.88701+0.039622*LN(ECM1)  +0.38964*LN(MBL2)+0.17720*LN(BTD)+0.077119*LN(RAB5C) |

ROC, receiver operating characteristic.

**Table S3. Gene ontology (GO) enrichment analysis of cellular components on among the identified 3171 tdEV proteins**

| **Index** | **GO ID** | **GO Term** | **P-value** | **Fold Enrichment** | **Bonferroni** | **Benjamini** | **FDR** |
| --- | --- | --- | --- | --- | --- | --- | --- |
| 1 | GO:0070062 | Extracellular exosome | 0 | 3.33298 | 0 | 0 | 0 |
| 2 | GO:0005829 | Cytosol | 2.05E-156 | 1.76426 | 2.43E-153 | 1.21E-153 | 9.73E-154 |
| 3 | GO:0005576 | Extracellular region | 1.40E-107 | 2.21931 | 1.66E-104 | 5.53E-105 | 4.43E-105 |
| 4 | GO:0005925 | Focal adhesion | 2.53E-99 | 3.96478 | 2.99E-96 | 7.49E-97 | 6.01E-97 |
| 5 | GO:0062023 | Collagen-containing extracellular matrix | 3.82E-80 | 3.72591 | 4.51E-77 | 9.03E-78 | 7.24E-78 |
| 6 | GO:0016020 | Membrane | 2.10E-69 | 1.54083 | 2.48E-66 | 4.13E-67 | 3.32E-67 |
| 7 | GO:0072562 | Blood microparticle | 3.89E-66 | 4.32501 | 4.60E-63 | 6.57E-64 | 5.27E-64 |
| 8 | GO:0005615 | Extracellular space | 2.39E-62 | 1.92134 | 2.83E-59 | 3.54E-60 | 2.84E-60 |
| 9 | GO:1904813 | Ficolin-1-rich granule lumen | 1.64E-47 | 4.96271 | 1.95E-44 | 2.16E-45 | 1.73E-45 |
| 10 | GO:0005737 | Cytoplasm | 1.75E-45 | 1.39205 | 2.06E-42 | 2.06E-43 | 1.66E-43 |

Table S4. Training results of the CNN-SVM algorithm with the output of weighting single and multiple EV protein markers using the LSBoost algorithm.

| EV protein markers | Accuracy (%) | AUC value | | | |
| --- | --- | --- | --- | --- | --- |
|  |  | **Normal** | **TNBC w/o recurrence** | **TNBC with recurrence** | **Other subtypes** |
| ECM1 (E) | 71.8 | 0.9419 | 0.8946 | 0.8669 | 0.8704 |
| MBL2 (M) | 70.7 | 0.9306 | 0.8803 | 0.8306 | 0.8890 |
| BTD (B) | 69.2 | 0.9094 | 0.8755 | 0.8250 | 0.8626 |
| RAB5C (R) | 66.3 | 0.8930 | 0.8568 | 0.8488 | 0.8109 |
| E+M | 77.2 | 0.9446 | 0.9115 | 0.9293 | 0.9082 |
| E+B | 76.9 | 0.9357 | 0.8872 | 0.9206 | 0.9178 |
| E+R | 76.4 | 0.9442 | 0.8939 | 0.9304 | 0.9193 |
| M+B | 75.1 | 0.9339 | 0.9047 | 0.9112 | 0.8794 |
| M+R | 74.4 | 0.9185 | 0.8929 | 0.9285 | 0.8976 |
| B+R | 73.9 | 0.9228 | 0.8805 | 0.8897 | 0.8551 |
| E+M+B | 82.9 | 0.9629 | 0.9513 | 0.9683 | 0.9360 |
| E+M+R | 80.6 | 0.9557 | 0.9602 | 0.9529 | 0.9223 |
| E+B+R | 79.5 | 0.9483 | 0.9395 | 0.9611 | 0.9395 |
| M+B+R | 78.3 | 0.9122 | 0.9204 | 0.8938 | 0.9087 |
| E+M+B+R | 84.7 | 0.9934 | 0.9481 | 0.9705 | 0.9576 |

AUC, area under the curve; TNBC, triple negative breast cancer; EV, extracellular vesicle; CNN, convolutional neural network; SVM, support vector machine.

Table S5. Diagnostic evaluation of multi-marker combinations

| Rank | Combination | Sensitivity | Specificity | AUC | SE | 95% Cl |
| --- | --- | --- | --- | --- | --- | --- |
| 1 | **ECM1+MBL2+BTD+RAB5C**  **(tdEV_protein_ score)** | 80.00% | 93.33% | 0.924 | 0.024 | 0.864 to 0.963 |
| 2 | ECM1+MBL2+RAB5C | 80.00% | 90.00% | 0.922 | 0.023 | 0.862 to 0.962 |
| 3 | ECM1+MBL2+BTD | 79.00% | 90.00% | 0.919 | 0.024 | 0.858 to 0.960 |
| 4 | ECM1+MBL2 | 73.00% | 90.00% | 0.918 | 0.023 | 0.856 to 0.959 |

* The rank 1 (in bold) was selected as the optimal combination and is called the ‘tdEV_protein_ score’.
